# The yeast Dbf4 Zn^2+^ finger domain suppresses single-stranded DNA at replication forks initiated from a subset of origins

**DOI:** 10.1007/s00294-022-01230-6

**Published:** 2022-02-11

**Authors:** Jeff Bachant, Elizabeth A. Hoffman, Chris Caridi, Constance I. Nugent, Wenyi Feng

**Affiliations:** 1 Department of Molecular Cell Systems Biology, University of California, Riverside, CA 92521 USA; 2 Department of Biochemistry and Molecular Biology, State University of New York Upstate Medical University, Syracuse, NY 13210 USA; 3 Department of Biological Sciences, University of Southern California, Los Angeles, CA 90089 USA

**Keywords:** Dbf4, DNA replication origin firing, DNA polymerase alpha, Primase, MCM

## Abstract

Dbf4 is the cyclin-like subunit for the Dbf4-dependent protein kinase (DDK), required for activating the replicative helicase at DNA replication origin that fire during S phase. Dbf4 also functions as an adaptor, targeting the DDK to different groups of origins and substrates. Here we report a genome-wide analysis of origin firing in a budding yeast mutant, *dbf4-zn*, lacking the Zn^2+^ finger domain within the C-terminus of Dbf4. At one group of origins, which we call dromedaries, we observe an unanticipated DNA replication phenotype: accumulation of single-stranded DNA spanning ± 5kbp from the center of the origins. A similar accumulation of single-stranded DNA at origins occurs more globally in *pri1-m4* mutants defective for the catalytic subunit of DNA primase and *rad53* mutants defective for the S phase checkpoint following DNA replication stress. We propose the Dbf4 Zn^2+^ finger suppresses single-stranded gaps at replication forks emanating from dromedary origins. Certain origins may impose an elevated requirement for the DDK to fully initiate DNA synthesis following origin activation. Alternatively, *dbf4-zn* may be defective for stabilizing/restarting replication forks emanating from dromedary origins during replication stress.

**Supplementary Information:**

The online version contains supplementary material available at 10.1007/s00294-022-01230-6.

## Introduction

Orderly genome duplication is achieved through a tightly regulated program of DNA replication origin (*ORI*) firing (Raghuraman and Brewer 2010; Marchal et al. 2019). *ORI*s in some nuclear regions fire early in S phase, while others fire late, leading to a defined spatiotemporal sequence for replicating chromosomes. An essential protein involved in controlling *ORI* firing is Dbf4, the activating subunit for Cdc7, the budding yeast Dbf4-dependent protein kinase (DDK; Jackson et al. 1993). Dbf4 is regulated through ubiquitin-mediated proteolysis in a cyclin-like manner, with Dbf4 accumulating in G_1_, peaking during the early S phase and declining at the metaphase to anaphase transition (Cheng et al. 1999; Weinreich and Stillman 1999; Godinho Ferreira et al. 2000). Activation of each *ORI* that fires during S phase requires *cis*-acting DDK phosphorylation of *ORI*-bound Mcm2-7 hexamers (Labib 2010). It has emerged that competition between *ORI*s for a limiting pool of active DDKis a key determinant of when and how efficiently different *ORI*s fire (Boos and Ferreira 2019).

Both positive- and negative-acting mechanisms modulate DDK access and activity towards *ORI*s. One important mechanism in budding yeast is that *ORI*s adjacent to centromeres (*CEN*s) gain preferential access to Dbf4, making *CEN ORI*s early and robustly firing *ORI*s (Raghuraman et al. 2001; Natsume et al. 2013). Dbf4 binds to the Ctf19/COMA kinetochore complex and is apparently off-loaded, increasing Dbf4 availability to proximal *ORI*s (Natsume et al. 2013). A second mechanism determining *ORI* early firing potential is controlled by the Forkhead transcription factors Fkh1 and Fkh2 (Knott et al. 2012). Fkh1/2 binds to specific chromatin regions, possibly forming chromosome domains that position *ORI*s to efficiently compete for Dbf4. Fkh1/2 also binds Dbf4, providing a mechanism for this competitive advantage (Fang et al. 2017). Conversely, *ORI*s can be delayed in firing through pathways acting in opposition to the DDK. One conserved mechanism involves Rif1 acting as a targeting factor for protein phosphatase 1 (PP1). Rif1/PP1 binds to particular *ORI*s and counteract Mcm2-7 phosphorylation, thereby conferring late firing timing (Hiraga et al. 2014; Dave et al. 2014; Mattarocci et al. 2014; Peace et al. 2014). Activation of the S phase checkpoint is an additional mechanism that delays *ORI* firing. In response to replication deterrents such as hydroxyurea (HU), the S phase checkpoint kinase Rad53 complexes with and extensively phosphorylates Dbf4, delaying activation of a large number of *ORI*s that fire later in the replication program (Lopez-Mosqueda et al. 2010; Zegerman and Diffley 2010; Chen et al. 2013; Almawi et al. 2016). The Rad53 check on *ORI* firing minimizes the number of stressed forks, contributing to fork stability at early *ORI*s. (Feng et al. 2006; Poli et al. 2012; Zhong et al. 2013).

Current evidence indicates the DDK acts uniformly at all *ORI*s to initiate firing. During G_1_, paired Mcm2-7 hexamers are loaded at licensed *ORI*s in an inactive configuration (Remus et al. 2009). DDK phosphorylation of Mcm4 and Mcm6 induces Mcm2-7 conformational changes that melt *ORI* DNA and allow the hexamers to encircle template DNA single strands (ssDNA) in the necessary configuration for bidirectional DNA synthesis (Li and O’Donnell 2018). DDK phospho-targeting of Mcm2-7 also generates binding sites for the Cdc45, Sld3 and Sld7 proteins (Deegan et al. 2016). In parallel, Cdk1 bound to S phase cyclins phosphorylates Sld2 and Sld3, inducing additional protein interactions leading to the assembly of the Cdc45, MCM, GINs (CMG) replicative helicase (Tanaka et al. 2007; Muramatsu et al. 2010). Ablation of an auto-inhibitory activity within the N-terminus of Mcm4 or a gain-of-function mutation in Mcm5 bypass the essential requirement for Dbf4 and Cdc7 (Hardy et al. 1997; Sheu and Stillman 2010). Thus, the minimal essential role for the DDK in DNA replication is to activate Mcm2-7. DDK bypass mutations exhibit sensitivity to replication inhibitors (Sheu and Stillman 2010), indicating the DDK mediates additional functions that optimize DNA replication or allow cells to tolerate replication stress.

Structurally, Dbf4 and its homologues in other eukaryotes (*e.g.,* Dbf4^hs^, Dbf4^mm^, and Chiffon^dm^) contain three conserved regions that mediate Dbf4 functions: motifs N, M and C (Masai and Arai 2000). Motif N contains a BRCT-like domain that binds Rad53 (Matthews et al. 2014), while motifs M and C are from separate interaction surfaces for Cdc7 (Dowell et al. 1994; Ogino et al. 2001). In particular, motif C contains a C2H2 Zn^2+^ finger domain that aligns motif C to contact Cdc7 (Hughes et al. 2012). In budding yeast, the entirety of motif C, including the Dbf4 Zn^2+^ finger, is not essential for cell growth, although the loss of the Zn finger leads to reduced DDK activity, temperature sensitivity, slow progression through S phase, and sensitivity to genotoxic stress (Harkins et al. 2009; Jones et al. 2010; Hughes et al. 2012). The basis for this spectrum of genome instability phenotypes is not well understood.

In a recent report, we analyzed genome-wide ssDNA replication intermediates in a *dbf4-zn* mutant lacking the Zn^2+^ finger domain (Julius et al. 2019). We found firing of *CEN ORI*s was strongly reliant on the Dbf4 Zn^2+^ finger. Here we extend our analysis of *dbf4-zn* to encompass other populations of *ORI*s. We identify a group of *ORI*s, which we call dromedary *ORI*s, that display an unanticipated replication defect in *dbf4-zn* in which ssDNA accumulates in the vicinity of *ORI*s. Based on the similarity of this phenotype to *rad53* mutants and *pri1-M4* mutants defective for the catalytic subunit of DNA primase, we suggest dromedary *ORI*s either impose an elevated requirement for the DDK to fully initiate DNA replication, or that the Dbf4 Zn^2+^ finger contributes to functions that maintain coupling between leading and lagging strand synthesis during replication stress.

## Materials and methods

### ssDNA mapping

Strain construction and methodology for generating genome-wide ssDNA datasets for wild type, *dbf4-zn*, *rad53-21*, *rad53-21 dbf4-zn* and *rad53 dbf-D3* strains have been previously described (Julius et al. 2019). For PRI and pri1-M4 ssDNA mapping, isogenic *PRI1* and *pri1-M4* cells of BY4741 background were grown in synthetic complete medium at 25 °C to logarithmic phase before synchronization. Cells were arrested in G1 by incubating with 3 µM alpha-factor for approximately 1.5 generations until unbudded cells reach at least 95%. Cells were then released from G1 arrest by the addition of 0.3 mg/ml pronase and allowed to enter S phase at 37 °C, the restrictive temperature for *pri1-M4* mutation. S phase samples collected at 30 min, along with G1 control samples collected before releasing into S phase, were embedded in agarose plugs and used for ssDNA mapping by microarrays as previously described (Feng et al. 2006, 2007). Briefly, agarose plugs containing the S phase samples from the *PRI1* and *pri1-M4* cells and their respective G1 controls were labeled for ssDNA via random-primed synthesis by Klenow (Exo-) at 37 °C for 1 h. Such a condition allows the labeling reaction to only occur on template DNA that contains single-stranded gaps, which are conducive to the incorporation of nucleotides without denaturation of the template DNA. The S phase DNA and G1 control were differentially labeled with dNTP mixes containing Cy3- and Cy5-dUTP, respectively, before co-hybridization onto the Agilent Yeast Whole Genome ChIP-to-chip 4 × 44 K (G4493A) microarrays. Fluorescence data were extracted by the Agilent Feature Extraction Software (v9.5.1). The relative quantity of ssDNA at a given genomic locus was calculated as the ratio of the fluorescent signal from the S phase sample to that of the G1 control, followed by Loess-smoothing over a 6-kb window at a step size of 250 bp.

### Calculation of peak amplitude/base ratios and AUC values from composite ssDNA replication profiles

Two methods were used to calculate profile peak to base ratios; results from both approaches are provided in Supplemental Table 2. In the first method, the base of the profile was defined as the distance between the minimum ssDNA values on either side of the *ORI* center. ssDNA maximum values on the left and right side of the profile (inclusive of the *ORI* center) were subtracted from the minimum values. Profile amplitude was defined as the average of the two differences.

The second approach utilized inflection points along the profile to determine amplitude to base ratios. For this approach, a one kbp sliding window was first used to smooth the profile data. The percent change between profile values at 250–300 bp intervals was then calculated. Using the positions of ssDNA maximum values (inclusive of the *ORI* center) as starting points, positions where the percent change of on either side of the profile flattened to 0.5% (or, for low amplitude profiles, 0.25%) were used to delimit the left and right sides of the major ssDNA feature within the profiles. Peak amplitude was defined as the average of ssDNA maximum values minus ssDNA inflection point values on either side of the *ORI*. Deconvolving the profiles in this fashion proved useful in defining the boundaries of profile features reflecting the accumulation of *ORI* ssDNA. Low amplitude profiles where 0.25% change, rather than 0.5% change, was used to identify major inflection points included: *rad53 dbf4-zn* 1 and 2 unchecked and checked *ORI*s, WT checked *ORI*s, *dbf4-zn* checked *ORI*s, *pri1-M4* checked *ORI*s.

AUC values were calculated as the sum of ssDNA values at each point within the meta-profile, extending between the two minimum values on either side of the *ORI* center.

## Results

### Meta-analyses of ssDNA replication profiles in rad53 dbf4 double mutants

We recently reported genome datasets for ssDNA replication intermediates in *dbf4* strains containing mutations affecting the C-terminus of the Dbf4 protein (Julius et al. 2019). Strains for these datasets were generated by transforming *dbf4-∆* or *rad53-21 dbf4-∆* cells with low copy plasmids expressing either *DBF4*, *dbf4-zn* or *dbf4-D3* under control of the endogenous promoter, referred to here as wild type (WT), *dbf4-zn*, *rad53-21*, *rad53-21 dbf4-zn* and *rad53 dbf-D3* strains. *dbf4-zn* is an internal deletion of amino acids 660–688 that form the Zn^2+^ finger within motif C. *dbf4-D3* contains a R701G mutation in a 26 amino acid C-terminal extension beyond the Zn^2+^ finger. *dbf4-zn* and *dbf4-D3* behave as recessive loss of function mutations, with *dbf4-zn* exhibiting more severe phenotypes than *dbf4-D3*. To generate ssDNA datasets, strains were arrested in G1 and released into media containing 200 mM HU at 30 °C. After 60 min ssDNA was isolated and hybridized to genome microarrays. ssDNA values from HU samples were normalized to the signal from G1 arrested cells, providing S/G1 ssDNA ratios for each position on the array.

**Fig. 1:**
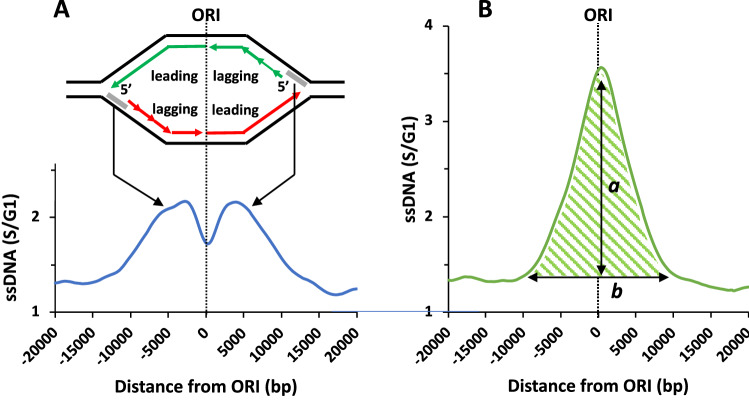
ssDNA meta-profiles and terminology. The terms meta-analyses/meta-profiles refer to the distribution of ssDNA associated with bidirectional replication forks emanating from a user-defined cohort of *ORI*s, slowed in their advance by HU nucleotide depletion. To generate meta-profiles, S/G1 values spanning ± 20 kbp of cohort *ORI*s are averaged and plotted relative to the composite *ORI* center. **A** Meta-profile for 146 early firing, unchecked *ORI*s in WT + HU. The terms early *ORI*s and late *ORI*s refer to relative firing timing in the S phrase program. Under standard laboratory conditions, *ORI*s that do or do not fire in 200 mM HU are stereotypical. However, since Rad53 can be activated throughout S phase, we use the terms unchecked and checked *ORI*s to indicate the delay in *ORI* firing is determined by the timing of Rad53 activation rather than an arbitrary point in S phase. In WT + HU, forks advance ~ 3000 bp prior to nucleotide depletion, corresponding with exposure of ~ 100 bp of ssDNA on the lagging template strand (grey line on replication bubble) (Sogo et al. 2002; Poli et al. 2012). This localized ssDNA signal produces a split peak meta-profile, with the shape and positioning of the peaks reflecting the distribution of forks in the cohort of *ORI*s (arrows) (Feng et al. 2006, 2007). Green and red represent newly synthesized Crick and Watson strands, respectively. Arrows indicate the direction of synthesis. Discontinuous synthesis of lagging strands is indicated by short arrows. **B** Corresponding meta-analysis for 146 unchecked *ORI*s in the *rad53* + HU dataset. Here the meta-profile resolves as a symmetrical peak of ssDNA centered over the *ORI* (Feng et al. 2006, 2009, 2011). Quantifiable parameters include the area under the curve (AUC, green shading), peak amplitude (a), and peak base (b). The ssDNA/base ratio (a/b) provides a metric for the extent to which ssDNA is biased towards the *ORI*. *CEN ORI*s and Fkh1/2 *ORI*s. These terms refer to categories of unchecked *ORI*s that differ in how they recruit the DDK to specify early firing potential. *CEN ORI*s are specified because microtubule attachments position them in a nuclear compartment with preferential access to the DDK. Fkh1/2 *ORI*s are specified through an ability of Fkh1/2 to recruit the DDK. Supplemental Table 1 specifies unchecked *ORI*s belonging to *CEN ORI* and Fkh1/2 *ORI* categories

The purpose of this current study was to perform a meta-analysis of *ORI* firing in the *dbf4-zn* mutant. A meta-analysis means ssDNA replication profiles for user-defined cohorts of *ORI*s are averaged, producing a composite meta-profile that can reveal emergent features of the data. Figure 1 provides information regarding the interpretation of meta-profiles and terminology. The pronounced accumulation of ssDNA at *ORI*s in *rad53* + HU (Fig. 1B) reflects: (1) formation of gapped replication bubbles due to uncoupling of leading and/or lagging strand synthesis in HU (Lopes et al. 2001; Sogo et al. 2002; Bermejo et al. 2011; Gan et al. 2017); (2) expansion of ssDNA gaps through exonuclease resection (Cotta-Ramusino et al. 2005); (3) defects in initiating lagging strand synthesis due to limiting Pol *α*/primase activity (Sogo et al. 2002); (4) reduced fork advance as a consequence of these defects. A schematic depicting abnormal *rad53* + HU fork structures that have been proposed in the literature is shown in Supplemental Fig. 1.

**Fig. 2:**
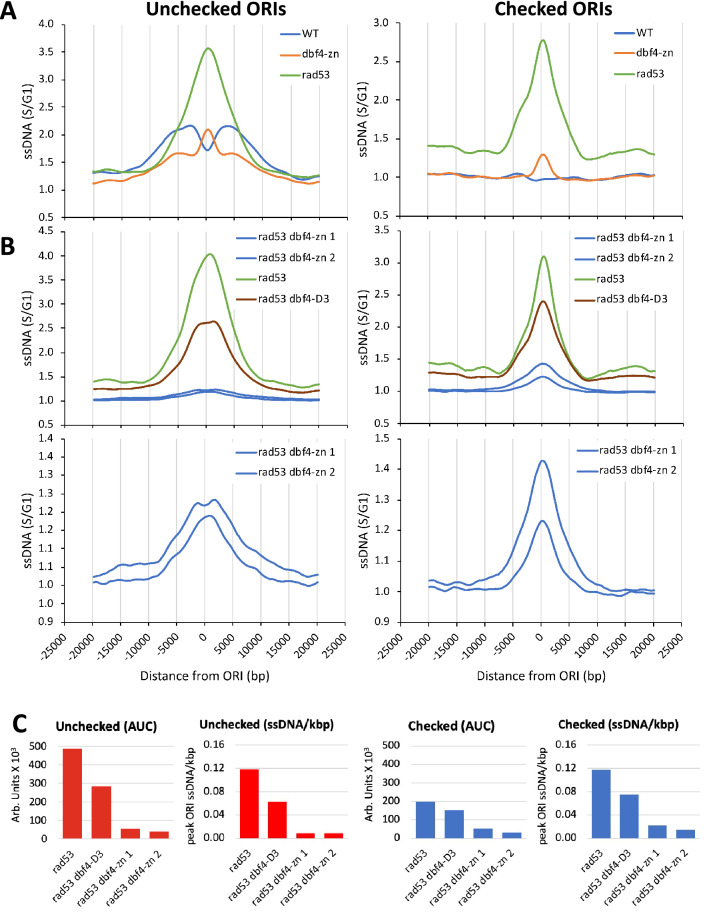
*ORI* ssDNA meta-profiles for unchecked and checked *ORI*s. **A** Comparison of *ORI* ssDNA meta-profiles in WT + HU, *dbf4-zn* + HU and *rad53* + HU datasets. All three strains were evaluated in the same experiment. Left panel displays meta-profiles generated from 146 unchecked *ORI*s (listed in Supplemental Table 1). Right panel displays corresponding meta-profiles generated from 186 checked *ORI*s that predominately fire only in the *rad53* + HU dataset. The *dbf4-zn* meta-composite for unchecked *ORI*s displays features of both the WT and *rad53* profiles. **B** Comparison of *ORI* ssDNA meta-profiles in *rad53* + HU and *rad53 dbf4* + HU datasets. Upper panels show meta-profiles for checked and unchecked *ORI*s generated from *rad53* + HU, *rad53 dbf4-D3* + HU and duplicate *rad53 dbf4-zn* + *HU* datasets, all evaluated within the same experiment. The two *rad53 dbf4-zn* + HU datasets were generated from independent strains. Lower panels show an expanded *y*-axis for *rad53 dbf4-zn* + HU meta-profiles. Note the flattening of ssDNA accumulation over unchecked *ORI*s in the *rad53 dbf4-D3* + HU and *rad53-dbf4-zn* + HU profiles. **C** Comparison of AUC and ssDNA accumulation (ssDNA/kbp) over unchecked (red) and checked (blue) *ORI* meta-composites in *rad53* + HU and *rad53 dbf4* + HU datasets. The presence of *dbf4-D3* and *dbf4-zn* in a *rad53* mutant reduces both utilization (AUC) and ssDNA accumulation (ssDNA/kbp)

As a first comparison, we examined whether the presence of *dbf4-zn* or *dbf4-D3* reduced *ORI* firing in HU-treated *rad53* mutants. We hypothesized such a reduction might occur if loss of Dbf4 compensated for the failure of Rad53 to inhibit Dbf4 in restraint of *ORI* firing. Figure 2A shows an alignment comparing meta-profiles for 146 unchecked *ORI*s in WT + HU, *rad53* + HU, and *dbf4-zn* + HU; WT + HU and *rad53* + HU meta-profiles are the same as shown in Fig. 1A and Fig. 1B for illustrative purposes. The checked *ORI* meta-profiles comprise a cohort of 186 additional *ORI*s activated in *rad53* + HU as previously defined in Julius et al. (2019). Several observations arise from these comparisons. First, reflecting the pronounced accumulation of *ORI* ssDNA in *rad53* + HU, the AUC of *rad53* + HU meta-profiles for unchecked and checked *ORI*s increased relative to WT (Fig. 2A, Supplemental Table 2). Second, the AUC for the *rad53* + HU unchecked *ORI* meta-profile is greater than the AUC for *rad53* + HU at checked *ORI*s, suggesting checked *ORI*s in *rad53* + HU are activated less efficiently (Fig. 2A, Supplemental Table 2). Third, *rad53* + HU ssDNA/base ratios are similar between unchecked and checked *ORI* meta-profiles. This suggests forks from checked and unchecked *ORI*s in *rad53* + HU experience similar forms of deregulation leading to accumulation of ssDNA. This is notable since unchecked and checked *ORI*s in *rad53* + HU fire before and after nucleotide depletion, respectively.

Figure 2B shows the corresponding unchecked and checked *ORI* meta-profiles for HU treated *rad53*, *rad53 dbf4-D3* and duplicate *rad53 dbf4-zn* strains. AUC measurements for both unchecked and checked *ORI* meta-profiles were decreased in *rad53 dbf4-D3* + HU and *rad53 dbf4-zn* + HU compared to *rad53* + HU controls, dramatically so for *rad53 dbf4-zn* (20X and 24X reductions; Fig. 2B, Supplemental Table 2). 71 of the 186 checked *ORI*s that fire in *rad53* + HU failed to fire in *rad53 dbf4-zn* + HU. A notable feature of *rad53 dbf4* + HU unchecked *ORI* meta-profiles was that reduced firing corresponded with a displacement of ssDNA further away from the *ORI* (Fig. 2B). This was most apparent for *rad53 dbf4-D3* + HU but could also be visualized with *rad53 dbf4-zn* + HU. The bottom graphs in Fig. 2B show an expanded *y*-axis of the same *rad53 dbf4-zn* + HU meta-profiles to better resolve this change in profile. In keeping with the displacement of the ssDNA signal away from *ORI*s, ssDNA/base ratios for *rad53 dbf4-D3* + HU and *rad53 dbf4-zn* + HU unchecked *ORI* meta-profiles were reduced 1.9X and 13.2X, respectively, compared to *rad53* + HU, while checked *ORI* ssDNA/base ratios were reduced 1.6X and 6.2X (Fig. 2C, Supplemental Table 2). This suggests reduced *ORI* utilization in *rad53 dbf4* mutants corresponds with a partial amelioration of *rad53* generating excess ssDNA over *ORI*s. Minimizing competition between forks may allow forks in *rad53 dbf4* mutants to progress further before they experience catastrophes.

### Phenotypic categories of ORIs affected by dbf4-zn

**Fig. 3:**
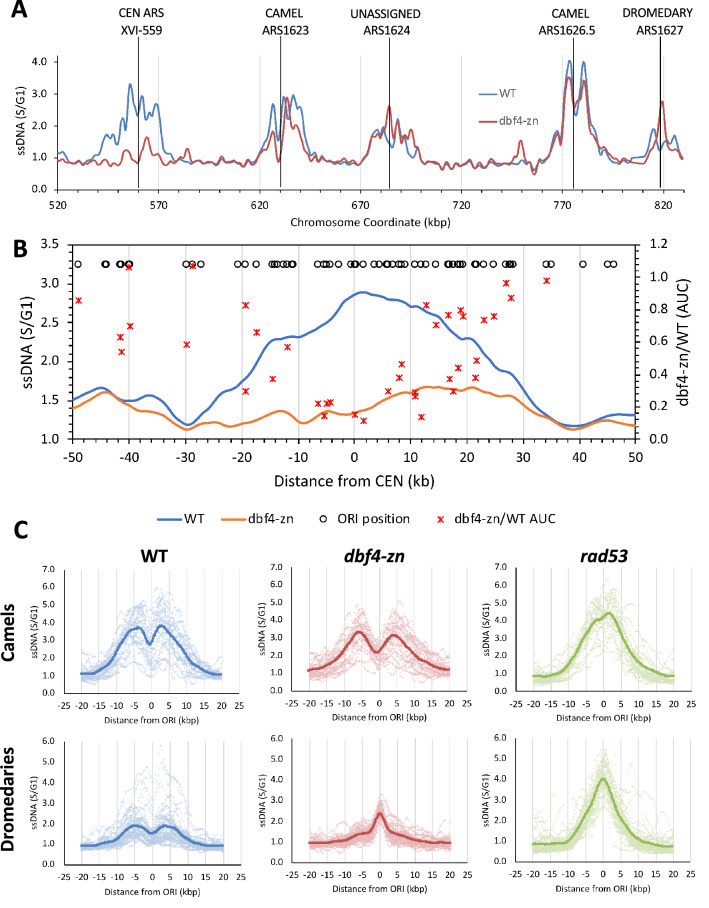
Evaluation of *CEN*-flanking, camel and dromedary *ORI*s in *dbf4-zn* mutants. **A** ssDNA profiles encompassing the right arm of chromosome 16 from WT + HU and dbf4-zn + HU datasets. Examples of *ORI* categories that are differentially affected by *dbf4-zn* are illustrated. **B** Meta-analysis of the effect of *dbf4-zn* on *CEN* regions. A ssDNA meta-profile for a ± 50 kpb region surrounding all 16 *CEN*s was generated from the WT + HU and *dbf4-zn* + HU datasets. Open circles at the top of the graph illustrate the location of all *ORI*s within this region throughout the genome. Solid lines show averaged ssDNA for WT + HU (blue) and *dbf4-zn* + HU (orange). Red X’s show the ratio of *dbf4-zn ORI* AUC values to WT *ORI* AUC values for 39 *CEN ORI*s where it was possible to plot individual *ORI* ssDNA profiles. In sum, the graph reveals a ± 20 kbp region where utilization of *CEN ORI*s and accumulation of ssDNA replication intermediates is greatly reduced by *dbf4-zn*. **C** Meta-profiles for camel and dromedary *ORI*s in WT + HU, *dbf4-zn* + HU and *rad53* + HU datasets. Upper panels, meta-profiles for camel *ORI*s. ssDNA meta-profiles were generated for a cohort of 29 *ORI*s that showed a two-humped, split peak ssDNA profile in *dbf4-zn* + HU. Solid lines display the averaged meta-profile; ssDNA profiles for individual *ORI*s are depicted as lighter dots. WT and *dbf4-zn* profiles for all 29 camel *ORI*s are displayed in Supplemental Fig. 2. Lower panels, meta-profiles for dromedary *ORI*s. 49 *ORI*s that displayed an atypical single hump of ssDNA over the *ORI* center were selected as a cohort for dromedary *ORI*s in the *dbf4-zn* + HU dataset. Solid lines display the averaged meta-profile; ssDNA profiles for individual *ORI*s are depicted as lighter dots. WT and *dbf4-zn* ssDNA profiles for all 49 dromedary *ORI*s are displayed in Supplemental Fig. 3

Several additional observations became apparent during our meta-analysis of *dbf4-zn* + HU. We previously found that the Dbf4 Zn^2+^ finger is required for robust firing of *CEN ORI*s (Julius et al. 2019). A meta-analysis of a ± 50 kbp window centered around all 16 *CEN*s provided a striking visualization of this phenotype (Fig. 3B). Four data series are shown in Fig. 3B. The first series (open circles) shows all unchecked *ORI*s within a ± 50 kbp *CEN* window, plotted at the top of the graph relative to *x*-axis kbp coordinates. This series reveals the high density of *ORI*s flanking *CEN*s throughout the genome. The second series is a *CEN*-centered ssDNA meta-profile for WT + HU (blue line, plotted relative to the left y-axis). This series reveals a broad accumulation of ssDNA replication intermediates associated with forks converging on *CEN*s. The third series is the corresponding ssDNA meta-profile for *dbf4*-*zn* + HU (orange line, plotted relative to the left *y*-axis). This shows that ssDNA accumulation at *CEN*s is largely eliminated in *dbf4-zn*. For the fourth series, WT + HU and *dbf4-zn* + HU datasets were inspected for *ORI*s with clearly delineated *ORI* profiles, and AUC values were determined. The ratios of individual *dbf4-zn*/WT AUCs were plotted (red X’s) relative to the right *y*-axis. 39 *ORI*s are depicted, revealing a prominent reduction in *ORI* utilization ± 20 kbp from the *CEN* in *dbf4-zn* + HU.

Unexpectedly, the unchecked *ORI* meta-profile for *dbf4-zn* + HU exhibited features of both the WT + HU and *rad53* + HU profiles. While there were split ssDNA peaks as in the WT + HU meta-profile, there was also an indication of a ssDNA peak centered over the *ORI*, reminiscent of *rad53* + HU (Fig. 2A). This led us to suspect that the *dbf4-zn* + HU composite was a combination of these two profiles. Inspection of individual *ORI*s in WT + HU and *dbf4-zn* + HU revealed this was indeed the case. Figure 3A shows a region of chromosome 16 where, for *dbf4-zn* + HU, two *ORI*s (ARS1623 and ARS1626.5) exhibit split peak profiles, one *ORI* (ARS1627) exhibits the unanticipated single ssDNA peak, one *CEN ORI* (XVI-559) were firing is strongly reduced, and one *ORI* (ARS1624) that is not readily assignable to split peak or single peak profiles. Since both split peak and single peak profiles were present in *dbf4-zn* + HU, we needed a convenient way to refer to them, and adopted the terms camel *ORI*s and dromedary *ORI*s, respectively, for this purpose. Camels are simply *ORI*s demonstrating the split peak ssDNA profile characteristic of normal fork advance in HU, while the form of dromedary *ORI*s suggests a novel component to the *dbf4-zn* + HU phenotype. To evaluate this possibility, we scored all 146 unchecked *ORI*s in *dbf4-zn* + HU and found ~ 75% of them could be assigned as either camel *ORI*s, dromedary *ORI*s, or *CEN ORI*s exhibiting reduced firing potential (Supplemental Table 1). 29 camel *ORI*s (Supplemental Fig. 2, Supplemental Table 1) and 49 dromedary *ORI*s (Supplemental Fig. 3, Supplemental Table 1) were selected as cohorts for meta-profile comparisons between *dbf4-zn* + HU, WT + HU and *rad53* + HU (Fig. 3C); these cohorts will be called camel *ORI*s and dromedary *ORI*s when referring to all three datasets. For *dbf4-zn* + HU, camel and dromedary *ORI* cohorts resolved with clearly distinct split peak and or single peak meta-profiles, respectively. WT + HU camel and dromedary cohorts both resolved with split peak meta-profiles, while *rad53* + HU camel and dromedary cohorts both resolved with single-peaked meta-profiles (Fig. 3C). Thus, the *dbf4-zn* + HU dromedary cohort represents a subset of unchecked *ORI*s that accumulate aberrant ssDNA like *rad53* + HU.

### Features of dbf4-zn + HU dromedary ORIs

**Fig. 4:**
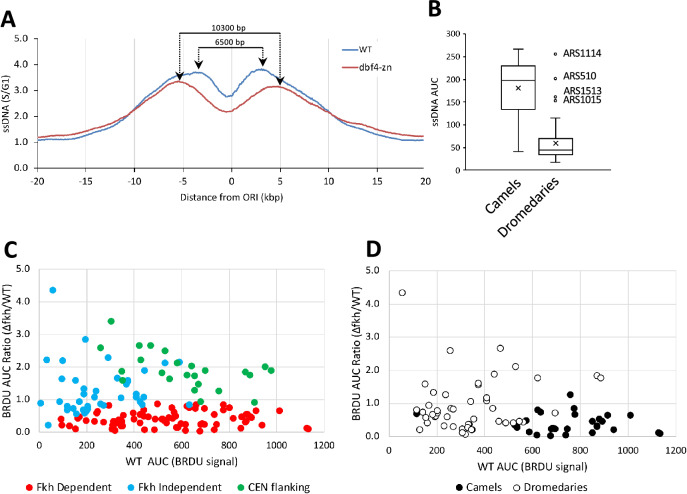
Characteristics of camel and dromedary *ORI*s. **A** Forks emanating from camel *ORI*s progress further in HU-treated *dbf4-zn* mutants compared to WT. Graph displays an overlay of the WT and *dbf4-zn* meta-profiles for camel *ORI*s. Arrows indicate maximal values for ssDNA peaks on either side of the *ORI* center. **B** Camel *ORI*s fire more efficiently than dromedaries. Box and whisker plots show the distribution of AUC values for all 29 camel and 49 dromedary *ORI*s in the WT + HU dataset. The four outliers (1.5 × inter-quartile value) are robustly utilized *CEN ORI*s in WT + HU but weakly firing dromedaries in *dbf4-zn* + HU. **C** Fkh1/2-dependence of different *ORI* populations. AUC values for BRDU incorporation at 143 *ORI*s were determined for *∆fkh1 ∆fkh2* + p*FKH2∆C* mutants and WT controls using the dataset published by Knott et al. (2012). The ratio of the *fkh1/2* mutant AUC to the WT AUC was calculated as a measurement of Fkh1/2-dependence. This ratio was then plotted as a function of the WT BRDU AUC for all 143 ORIs. Green circles are *CEN ORI*s, which are largely Fkh1/2-independent in their firing potential. Red circles are *ORI*s that by the criteria of Knott et al. (2012) were identified as Fkh1/2-dependent *ORI*s. Blue circles are the remaining Fkh1/2-independent group of *ORI*s; these tend to fire less robustly in WT. Note that some Fkh1/2-independent *ORI*s show increased utilization in the *fkh* mutant (*fkh*/WT ratios > 1). **D** The identity of camel (black circles) and dromedary (open circles) *ORI*s was superimposed on the graph shown in C. Camel *ORI*s correspond with robustly firing *ORI*s that are strongly dependent on the Fkh1/2 pathway

We next asked what features distinguish the dromedary *ORI* cohort. As a first observation, it was apparent that forks from *dbf4-zn* + HU camel *ORI*s progressed further, on average than forks from WT + HU camel *ORI*s (Fig. 4A). One explanation is that reduced *CEN ORI* firing in *dbf4-zn* + HU increases dNTP availability to forks from other *ORI*s (Poli et al. 2012; Zhong et al. 2013). As a second observation, the average AUC for WT + HU camel *ORI*s was ~ 3 time greater than the average AUC for WT + HU dromedary *ORI*s (Fig. 4B, *p* < 0.001, Student’s *t* test). The AUC distribution for WT + HU dromedary *ORI*s contained four high-end outliers, all of which were *CEN ORI*s greatly reduced in firing potential in *dbf4-zn* + HU. This suggests that camel *ORI*s tend to fire more efficiently than dromedary *ORI*s in WT + HU cells.

We asked if local genome elements are known to alter *ORI* activity, including tRNAs, transposable elements, RNA: DNA hybrids and relative orientation of replication and transcription (Voytas and Boeke 1993; Hoffman et al. 2015; Costantino and Koshland 2018) differentiated camel and dromedary *ORI*s, but no significant difference in the association was observed (Supplemental Table 3). However, we did observe a higher nucleosome accessibility in camel *ORI*s compared to dromedaries based on nucleoATAC scores (Schep et al. 2015), average score 2.62 vs. 0.56, *p* = 0.14, Student’s *t* Test, one-tailed distribution, equal variance). This suggests camels reside in a more open chromatin environment conducive to *ORI* activation.

We also examined whether camel and dromedary *ORI* differed in their reliance on the Fkh1/2 pathway for early firing potential. To do this, we utilized the dataset published by Knott et al. (2012) to calculate a ratio between the AUC for BRDU incorporation in a *∆fkh1 ∆fkh2* + p*FKH2∆C* mutant and the AUC for BRDU incorporation in their WT control for 143 *ORI*s described in their study. In Fig. 4C the set of *∆fkh1 ∆fkh2* + p*FKH2∆C*/WT ratios are plotted as a function of the corresponding WT BRDU AUC. *ORI*s that, by the criteria of Knott et al. (2012), were evaluated as Fkh1/2-dependent (red circles) or Fkh1/2-independent (blue circles) are indicated. *CEN ORI*s are also plotted as a separate Fkh1/2-independent category (green circles). The identity of camel and dromedary *ORI*s was then superimposed (Fig. 4D), revealing camel *ORI*s are almost uniformly Fkh1/2-dependent *ORI*s. Of the 29 *ORI*s selected as camels (filled circles), 27 (93%) were Fkh1/2-dependent. In contrast, dromedary *ORI*s (open circles) tended to be Fkh1/2-independent, although this correlation was not as predictive (30 of 49 dromedaries were Fkh1/2-independent). To summarize, camel *ORI*s are characterized by Fkh1/2-dependent recruitment of Dbf4, an open chromatin environment, and split peak ssDNA replication profiles in *dbf4-zn* + HU. Dromedary *ORI*s, in contrast, tend to lack *CEN*- or Fkh1/2-specified DDK recruitment, corresponding with reduced firing potential and accumulation of *ORI* ssDNA in *dbf4-zn* + HU.

### dbf4-zn and pri1-M4 mutants show a similar ssDNA profile at dromedary ORIs

**Fig. 5:**
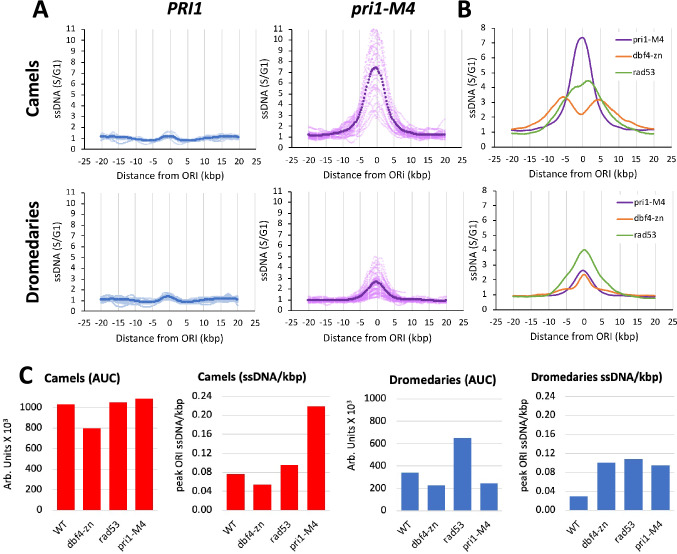
Similarities between *dbf4-zn* and *pri1-M4* mutants at dromedary *ORI*s. **A** Meta-analysis of camel and dromedary *ORI*s in *pri1-M4* mutants. Genome-wide ssDNA replication profiles were generated for *pri1-M4* mutants and an isogenic WT control (*PRI1*) at a *pri1-M4* non-permissive temperature of 37 °C. Graphs display ssDNA meta-profiles (solid lines) and profiles for individual *ORI*s (lighter data points) for 29 camel and 49 dromedary *ORI*s from the *pri1-M4* dataset. *pri1-M4* cells display accumulation of ssDNA over both camel and dromedary *ORI*s. *PRI1* controls in this experiment have largely completed S phase at the time of sampling, producing meta-profiles that are close to baseline values. This is because HU was not included in the experiment to reproduce the conditions under which *pri1-M4* and *rad53* + HU were initially compared in the work of Sogo et al. (2002). **B** Overlays comparing *dbf4-zn* + HU, *rad53* + HU and *pri1-M4* meta-profiles for camel and dromedary *ORI*s. Note the superimposition of the *dbf4-zn* + HU and *pri1-M4* dromedary meta-profiles. **C** Comparison of AUC and ssDNA accumulation (ssDNA/kbp) over camel (red) and dromedary (blue) *ORI* meta-profiles in WT + HU, *rad53* + HU, *dbf4-zn* + HU and *pri1-M4* datasets. *rad53* + HU *dbf4-zn* + HU and *pri1-M4* cells show a quantitatively similar tendency to accumulate ssDNA at *ORI*s

Finally, we considered the nature of the *dbf4-zn* + HU defect leading to ssDNA accumulation at dromedary *ORI*s. Previously, defective replication fork structures in *rad53* + HU have been compared to *pri1-M4* mutants defective for the catalytic subunit of DNA primase (Marini et al. 1997; Sogo et al. 2002). Remarkably similar single-stranded replication bubbles were visualized in both mutants, leading to the suggestion that *rad53* + HU and *pri1-M4* might share a common defect in lagging strand synthesis (Sogo et al. 2002). Accordingly, we generated a new ssDNA dataset for *pri1-M4*, releasing cells from G1 at a *pri1-M4* non-permissive temperature of 37 °C. To parallel the conditions of Sogo et al. 2002 as closely as possible, we note the *pri1-M4* strain was not treated with HU. Meta-analysis of the *pri1-M4* camel cohort of *ORI*s showed a dramatic accumulation of *ORI*-centered ssDNA (Fig. 5A), with a ssDNA/base ratio 2.3 ×  higher than *rad53* + HU (Fig. 5B and 5C, Supplemental Table 2). *pri1-M4* mutants also accumulated a smaller peak of *ORI* ssDNA in the meta-profile for the dromedary cohort of *ORI*s (Fig. 5A). Remarkably, the *pri1-M4* dromedary meta-profile was largely superimposable upon the dromedary meta-profile of *dbf4-zn* + HU (Fig. 5B), with AUC and ssDNA peak/base ratios that were quite similar between the two strains (Fig. 5C). Thus, qualitatively and quantitatively, the *dbf4-zn* + HU ssDNA profile at dromedary *ORI*s closely resembles the profile observed following a reduction in DNA primase activity.

## Discussion

In this report, we performed a genome-wide analysis of *ORI* firing in a *dbf4-zn* mutant lacking the C-terminal Zn finger domain, focusing on 146 unchecked *ORI*s that fire in S phase checkpoint proficient cells. A principal new finding is that 49 of these *ORI*s, which we call dromedary *ORI*s, display an aberrant ssDNA replication profile in *dbf4-zn* + HU reminiscent of *rad53* + HU and *pri1-M4* mutants. Both *rad53* + HU and *pri1-M4* form DNA replication bubbles containing extensive single-stranded gaps (Sogo et al. 2002), which likely determine their ssDNA replication profiles. Thus, rather than an “all or none” firing defect, the effect of *dbf4-zn* at dromedary *ORI*s would appear to be a perturbation to DNA synthesis that leads to single-stranded gaps in replication forks. To our knowledge, this is a novel phenotype for a budding yeast *dbf4* mutant. Defective DNA replication forks may underlie genome instability phenotypes previously associated with loss of the Dbf4 Zn finger domain in yeast, including slow progression through S phase, accumulation of DNA damage and sensitivity to forms of genotoxic stress including HU (Harkins et al. 2009; Jones et al. 2010; Hughes et al. 2012; Julius et al. 2019). The questions become what causes ssDNA gaps to accumulate at forks in *dbf4-zn* + HU, why is this defect specific to particular *ORI*s and what insights does the dromedary phenotype provide into Dbf4 function?

A consideration of aberrant replication fork structures in *rad53* + HU and *pri1-M4* mutants is likely to be informative regarding the *dbf4-zn* + HU dromedary phenotype. An early study examined replication bubbles in *rad53* + HU and *pri1-M4* mutants by electron microscopy, revealing extensively gapped forks and hemi-replicated bubbles (*i.e.* one side of the bubble completely double-stranded and the other side completely single-stranded) in both strains (Sogo et al. 2002). To account for this, it was proposed firing of unchecked *ORI*s in *rad53* + HU elevated the catalytic demand on Polαprimase, leading to a failure to initiate or maintain lagging strand synthesis (Supplemental Fig. 1B, D, F). More recent evidence suggests an additional role for Rad53 in maintaining a functional coupling between CMG and Polεat the site of leading strand synthesis, with failure to restrain CMG/Polε in *rad53* + HU exposing unwound ssDNA on the leading strand template (Gan et al. 2017; Devbhandari and Remus 2020) (Supplemental Fig. 1C). Notably, exonuclease resection of nascent leading or lagging strands arising from initiation of elongation defects in *rad53* + HU or *pri1-M4* mutants could extend ssDNA gaps towards *ORI*s, potentially generating hemi-replicated bubbles (Sogo et al. 2002; Cotta-Ramusino et al. 2005; Feng et al. 2006) (Supplemental Fig. 1E). Hemi-replicated bubbles could also arise directly through defective initiation of DNA synthesis, for example, initiation defects producing unidirectional forks (Sogo et al. 2002)(Supplemental Fig. 1F). Thus, while defective initiation of lagging strand synthesis likely accounts for the ssDNA replication profile associated with *pri1-M4*, *rad53* + HU may experience a broader range of fork defects, potentially differentially affecting *ORI*s that fire before or after nucleotide depletion. Thus, the *dbf4-zn* dromedary phenotype seems likely to arise through initiation or elongation defects in fork structure.

The molecular defect leading to the *dbf4-zn* dromedary phenotype is not resolved by our study. As laid out in the Introduction, however, the best-understood role for the DDK is to activate paired Mcm2-7 hexamers to encircle melted template strands in a configuration supporting bi-directional fork movement. DDK phosphorylation of Mcm2-7 is also necessary to recruit initiation factors for CMG assembly. Recent evidence suggests the multiplicity of DDK phosphorylation on Mcm2-7 corresponds with the extent of initiation factor recruitment, with more robustly phosphorylated subunits potentiating a later acting, rate limiting, step in CMG assembly (De Jesús-Kim et al. 2021). Other DDK regulatory circuits, such as a role for Mcm10 in DDK phosphorylation of Mcm2, have also been proposed, potentially stimulating RPA and Polα loading at *ORI*s (Walter and Newport 2000; Zhu et al. 2007; Perez-Arnaiz et al. 2017). Our analysis suggests a distinguishing feature of dromedary *ORI*s is that they lack known (*CEN*- or Fkh1/2-directed) Dbf4 enrichment mechanisms. Dromedary *ORI*s in *dbf4-zn* + HU may therefore fire with comparatively reduced Mcm2-7 phospho-targeting, allowing hexamers to pass on opposing strands but not completely supporting subsequent events in CMG/replisome assembly or initiation of DNA synthesis.

With respect to post-initiation forms of regulation by the DDK, accumulating evidence, mostly in animal cells, suggests continued DDK activity is necessary for fork advance during replication stress (Dolson et al. 2021). Roles for the DDK appear to include regulation of fork reversals, nuclease processing, and gap filling as a means to restart stalled forks (Sasi et al. 2018; Rainey et al. 2020; Jones et al. 2021; Cabello-Lobato et al. 2021). Additionally, continued DDK phosphorylation of Mcm2-7 (Bastia et al. 2016; Alver et al. 2017), and potentially phosphorylation of fork pausing factors (Murakami and Keeney 2014), may enforce polymerase coupling at stalled forks. We, therefore, speculate incomplete DDK phosphorylation of Mcm2-7 or other substrates in *dbf4-zn* could predispose forks from dromedary *ORI*s to become destabilized in HU. Another factor that may function with the DDK is the Stn1 protein, a component of the CST telomere binding complex (Grandin et al. 1997). Previous work in yeast and human cells indicates Stn1 stimulates *ORI* firing under conditions of replication stress and physically interacts with Polα/primase, Mcm2 and Mcm7 (Gasparyan et al. 2009; Wang et al. 2019). In an accompanying paper, we present evidence that yeast Stn1 may stimulate DDK towards Mcm2-7 and that Stn1 abrogation leads to the accumulation of ssDNA at non-telomeric chromosomal regions. We are currently investigating the possibility that Stn1 acts in concert with the DDK to facilitate Mcm2-7 function in *ORI* firing or in replication fork integrity during replication stress.

## Supplementary Information

Below is the link to the electronic supplementary material.

Supplementary file1 (DOCX 44 KB)

Supplementary file2 (TIFF 2024 KB)

Supplementary file3 (TIFF 9831 KB)

Supplementary file4 (TIFF 2786 KB)

Supplementary file5 (TIFF 8522 KB)

Supplementary file6 (TIFF 1336 KB)
