# Supplementary material for: The yeast Dbf4 Zn2+ finger domain suppresses single-stranded DNA at replication forks initiated from a subset of origins
Source: Curr Genet. 2022 Feb 11;68(2):253–65. doi: 10.1007/s00294-022-01230-6 (PMC8976809; doi:10.1007/s00294-022-01230-6)
Supplement: Supplementary file 1 — Supplementary file1 (DOCX 44 KB) [file 294_2022_1230_MOESM1_ESM.docx]

**SUPPLEMENTAL**

**Supplemental Table 1.** Attributes of unchecked cohort of *ORI*s.

| \| Xsome \| Position^1^ \| Name \| ∆ ∆ to *CEN^2^* \| Fire WT^3^ \| Fire *dbf4-zn^4^* \| AUC ratio^5^ \| Assignment^6^ \| \| --- \| --- \| --- \| --- \| --- \| --- \| --- \| --- \| \| 1 \| 124 \| ARS107 \| -27051 \| Y \| N \|  \| UA \| \| 1 \| 137 \| ARS107.5 \| -14125 \| Y \| N \|  \| UA ,CEN \| \| 1 \| 147 \| ARS108 \| -4329 \| Y \| Y \| 0.23 \| UA ,CEN \| \| 1 \| 160 \| ARS109 \| 8492 \| Y \| Y \| 0.46 \| UA ,CEN \| \| 1 \| 166 \| I-166 \| 14475 \| Y \| Y \| 0.71 \| UA ,CEN \| \| 1 \| 176 \| ARS110 \| 24753 \| Y \| Y \| 0.76 \| CAMEL, FKH \| \| 1 \| 198 \| I-198 \| 46215 \| Y \| N \|  \| UA \| \| 2 \| 63 \| ARS202 \| -174964 \| Y \| Y \| 0.86 \| CAMEL, FKH \| \| 2 \| 198 \| ARS207.5 \| -39954 \| Y \| Y \| 1.07 \| DROMEDARY \| \| 2 \| 210 \| ARS207.8 \| -28642 \| Y \| Y \| 1.07 \| UA ,FKH \| \| 2 \| 238 \| ARS208 \| -506 \| Y \| Y \| 0.65 \| UA ,CEN \| \| 2 \| 255 \| ARS209 \| 16746 \| Y \| Y \| 0.77 \| UA ,CEN \| \| 2 \| 326 \| ARS211 \| 87950 \| Y \| Y \| 1.07 \| DROMEDARY \| \| 2 \| 408 \| ARS214 \| 169681 \| Y \| Y \| 0.90 \| UA \| \| 2 \| 487 \| ARS216 \| 248518 \| Y \| Y \| 1.19 \| DROMEDARY, FKH \| \| 2 \| 623 \| ARS220 \| 384493 \| Y \| Y \| 0.76 \| DROMEDARY, FKH \| \| 2 \| 632 \| ARS221 \| 393823 \| Y \| Y \| 0.94 \| UA ,FKH \| \| 3 \| 39 \| ARS305 \| -75009 \| Y \| Y \| 0.79 \| CAMEL, FKH \| \| 3 \| 75 \| ARS306 \| -39874 \| Y \| Y \| 0.70 \| CAMEL, FKH \| \| 3 \| 109 \| ARS307 \| -5408 \| Y \| Y \| 0.15 \| UA ,CEN \| \| 3 \| 115 \| ARS308 \| 183 \| Y \| Y \| 0.15 \| UA ,CEN \| \| 3 \| 132 \| ARS309 \| 17709 \| Y \| Y \| 0.30 \| UA ,CEN \| \| 3 \| 167 \| ARS310 \| 52476 \| Y \| Y \| 0.89 \| DROMEDARY \| \| 3 \| 225 \| ARS315 \| 110489 \| Y \| Y \| 0.73 \| UA \| \| 4 \| 330 \| ARS413 \| -120075 \| Y \| Y \| 0.87 \| UA ,FKH \| \| 4 \| 408 \| ARS414 \| -41572 \| Y \| Y \| 0.63 \| DROMEDARY \| \| 4 \| 435 \| ARS415 \| -14541 \| Y \| Y \| 0.38 \| UA ,CEN \| \| 4 \| 463 \| ARS416 \| 12802 \| Y \| Y \| 0.83 \| UA ,CEN \| \| 4 \| 477 \| IV-477 \| 27027 \| Y \| Y \| 0.96 \| UA \| \| 4 \| 484 \| ARS417 \| 34206 \| Y \| Y \| 0.98 \| DROMEDARY \| \| 4 \| 505 \| ARS417.5 \| 55694 \| Y \| Y \| 1.03 \| DROMEDARY \| \| 4 \| 555 \| ARS418 \| 105580 \| Y \| Y \| 0.83 \| UA ,FKH \| \| 4 \| 568 \| ARS419 \| 117851 \| Y \| Y \| 0.90 \| UA ,FKH \| \| 4 \| 879 \| IV-879 \| 429528 \| Y \| Y \| 0.80 \| UA \| \| 4 \| 899 \| IV-899 \| 449307 \| Y \| Y \| 1.11 \| UA \| \| 4 \| 914 \| ARS428 \| 464142 \| Y \| Y \| 0.78 \| CAMEL, FKH \| \| 4 \| 1017 \| ARS430 \| 567010 \| Y \| Y \| 0.90 \| DROMEDARY \| \| 4 \| 1159 \| ARS432 \| 709612 \| Y \| Y \| 1.05 \| UA ,FKH \| \| 4 \| 1166 \| ARS432.5 \| 716347 \| Y \| Y \| 0.97 \| UA ,FKH \| \| 5 \| 59 \| ARS507 \| -92645 \| Y \| Y \| 0.81 \| UA ,FKH \| \| 5 \| 94 \| ARS508 \| -57947 \| Y \| Y \| 0.83 \| CAMEL, FKH \| \| 5 \| 146 \| ARS510 \| -6384 \| Y \| Y \| 0.23 \| CEN  DROMEDARY \| \| 5 \| 174 \| ARS511 \| 21711 \| Y \| Y \| 0.49 \| UA , CEN \| \| 5 \| 288 \| ARS514 \| 135583 \| Y \| Y \| 0.87 \| DROMEDARY \| \| 5 \| 302 \| ARS514.5 \| 149769 \| Y \| N \|  \| UA \| \| 5 \| 317 \| V-317 \| 164631 \| Y \| Y \| 1.38 \| DROMEDARY \| \| 5 \| 354 \| ARS516 \| 201584 \| Y \| Y \| 0.75 \| CAMEL, FKH \| \| 5 \| 407 \| ARS517 \| 254804 \| Y \| Y \| 1.04 \| DROMEDARY \| \| 5 \| 439 \| ARS518 \| 287010 \| Y \| Y \| 0.90 \| DROMEDARY \| \| 5 \| 499 \| ARS520 \| 346836 \| Y \| N \|  \| UA \| \| 6 \| 119 \| ARS603.5 \| -29772 \| Y \| Y \| 0.58 \| DROMEDARY \| \| 6 \| 136 \| ARS605 \| -12534 \| Y \| N \|  \| UA ,CEN \| \| 6 \| 168 \| ARS606 \| 19261 \| Y \| Y \| 0.76 \| CEN CAMEL \| \| 6 \| 199 \| ARS607 \| 50875 \| Y \| Y \| 0.73 \| CAMEL, FKH \| \| 6 \| 217 \| ARS608 \| 67955 \| Y \| Y \| 1.28 \| UA ,FKH \| \| 7 \| 163 \| ARS707 \| -333670 \| Y \| N \|  \| UA \| \| 7 \| 204 \| ARS710 \| -292945 \| Y \| Y \| 0.91 \| DROMEDARY, FKH \| \| 7 \| 286 \| ARS714 \| -210885 \| Y \| Y \| 0.56 \| DROMEDARY \| \| 7 \| 389 \| ARS717 \| -108208 \| Y \| Y \| 0.75 \| CAMEL, FKH \| \| 7 \| 421 \| ARS718 \| -75766 \| Y \| Y \| 0.85 \| UA ,FKH \| \| 7 \| 485 \| ARS719 \| -11937 \| Y \| Y \| 0.57 \| UA ,CEN \| \| 7 \| 509 \| ARS720 \| 11871 \| Y \| Y \| 0.14 \| UA ,CEN \| \| 7 \| 575 \| ARS722 \| 77786 \| Y \| Y \| 0.78 \| UA \| \| 7 \| 660 \| ARS727 \| 162949 \| Y \| Y \| 1.73 \| DROMEDARY \| \| 7 \| 715 \| ARS728 \| 218432 \| Y \| Y \| 0.54 \| UA ,FKH \| \| 7 \| 778 \| ARS729 \| 281109 \| Y \| Y \| 0.79 \| UA ,FKH \| \| 7 \| 835 \| ARS731 \| 337631 \| Y \| Y \| 0.83 \| UA ,FKH \| \| 7 \| 888 \| ARS731.5 \| 391492 \| Y \| Y \| 0.78 \| CAMEL, FKH \| \| 8 \| 64 \| ARS805 \| -41267 \| Y \| Y \| 0.54 \| UA \| \| 8 \| 112 \| ARS805.7 \| 5891 \| Y \| N \|  \| UA ,CEN \| \| 8 \| 116 \| VIII-116 \| 10831 \| Y \| Y \| 0.29 \| UA \| \| 8 \| 133 \| ARS807 \| 27830 \| Y \| Y \| 0.87 \| CAMEL, FKH \| \| 8 \| 297 \| ARS815 \| 191540 \| Y \| Y \| 1.00 \| CAMEL, FKH \| \| 8 \| 448 \| ARS820 \| 342097 \| Y \| Y \| 0.50 \| DROMEDARY \| \| 9 \| 74 \| ARS907 \| -281673 \| Y \| Y \| 1.65 \| DROMEDARY \| \| 9 \| 106 \| ARS909 \| -249750 \| Y \| Y \| 0.83 \| UA ,FKH \| \| 9 \| 215 \| ARS913 \| -140934 \| Y \| Y \| 0.85 \| CAMEL, FKH \| \| 9 \| 246 \| ARS913.5 \| -109871 \| Y \| Y \| 1.51 \| DROMEDARY \| \| 9 \| 342 \| ARS919 \| -13710 \| Y \| N \|  \| UA ,CEN \| \| 9 \| 357 \| ARS920 \| 1591 \| Y \| Y \| 0.12 \| UA ,CEN \| \| 9 \| 407 \| IX-407 \| 50941 \| Y \| Y \| 0.81 \| CAMEL, FKH \| \| 10 \| 68 \| ARS1005 \| -368652 \| Y \| Y \| 1.40 \| DROMEDARY \| \| 10 \| 100 \| ARS1006 \| -336783 \| Y \| Y \| 1.22 \| CAMEL \| \| 10 \| 204 \| ARS1008 \| -232189 \| Y \| Y \| 0.71 \| UA ,FKH \| \| 10 \| 228 \| ARS1009 \| -207866 \| Y \| Y \| 0.92 \| UA \| \| 10 \| 376 \| ARS1013 \| -60698 \| Y \| Y \| 0.80 \| CAMEL \| \| 10 \| 417 \| ARS1014 \| -19349 \| Y \| Y \| 0.83 \| CEN, CAMEL, FKH \| \| 10 \| 442 \| ARS1015 \| 6093 \| Y \| Y \| 0.30 \| CEN  DROMEDARY \| \| 10 \| 455 \| ARS1016 \| 18402 \| Y \| Y \| 0.44 \| UA ,CEN \| \| 10 \| 459 \| ARS1017 \| 22949 \| Y \| Y \| 0.74 \| UA \| \| 10 \| 540 \| ARS1018 \| 103997 \| Y \| Y \| 0.79 \| CAMEL, FKH \| \| 10 \| 613 \| ARS1019 \| 176399 \| Y \| Y \| 0.78 \| CAMEL, FKH \| \| 10 \| 684 \| ARS1021 \| 247213 \| Y \| Y \| 0.85 \| CAMEL, FKH \| \| 11 \| 56 \| ARS1103 \| -384037 \| Y \| Y \| 0.91 \| CAMEL, FKH \| \| 11 \| 153 \| ARS1106 \| -286777 \| Y \| Y \| 1.14 \| DROMEDARY \| \| 11 \| 258 \| ARS1107 \| -182216 \| Y \| N \|  \| UA \| \| 11 \| 302 \| ARS1107.5 \| -137492 \| Y \| Y \| 0.59 \| UA \| \| 11 \| 329 \| ARS1109 \| -110384 \| Y \| Y \| 0.75 \| DROMEDARY \| \| 11 \| 448 \| ARS1114 \| 7945 \| Y \| Y \| 0.38 \| CEN  DROMEDARY \| \| 11 \| 457 \| ARS1114.5 \| 16995 \| Y \| Y \| 0.38 \| UA, CEN \| \| 11 \| 517 \| ARS1116 \| 76948 \| Y \| Y \| 1.05 \| DROMEDARY \| \| 12 \| 92 \| ARS1206 \| -59348 \| Y \| Y \| 0.71 \| UA, FKH \| \| 12 \| 93 \| ARS1206.5 \| -57474 \| Y \| Y \| 0.72 \| UA \| \| 12 \| 140 \| XII-140 \| -10886 \| Y \| N \|  \| UA, CEN \| \| 12 \| 157 \| ARS1209 \| 5879 \| Y \| N \|  \| UA, CEN \| \| 12 \| 231 \| ARS1211 \| 80415 \| Y \| Y \| 0.83 \| CAMEL, FKH \| \| 12 \| 373 \| ARS1213 \| 222392 \| Y \| Y \| 0.77 \| UA, FKH \| \| 12 \| 413 \| ARS1215 \| 261897 \| Y \| Y \| 0.59 \| DROMEDARY \| \| 12 \| 513 \| ARS1217 \| 362107 \| Y \| Y \| 0.53 \| UA, FKH \| \| 12 \| 603 \| ARS1218 \| 452161 \| Y \| Y \| 0.69 \| UA, FKH \| \| 12 \| 660 \| ARS1220 \| 509062 \| Y \| Y \| 0.62 \| DROMEDARY \| \| 12 \| 731 \| ARS1222 \| 579668 \| Y \| Y \| 0.46 \| UA \| \| 12 \| 745 \| ARS1223 \| 594175 \| Y \| Y \| 0.60 \| DROMEDARY, FKH \| \| 12 \| 794 \| ARS1226 \| 643259 \| Y \| N \|  \| UA \| \| 13 \| 32 \| ARS1303 \| -236279 \| Y \| Y \| 0.92 \| CAMEL, FKH \| \| 13 \| 94 \| ARS1305 \| -173751 \| Y \| Y \| 1.02 \| DROMEDARY \| \| 13 \| 137 \| ARS1307 \| -130667 \| Y \| Y \| 0.83 \| DROMEDARY \| \| 13 \| 159 \| ARS1307.5 \| -109028 \| Y \| Y \| 1.70 \| UA \| \| 13 \| 184 \| ARS1308 \| -84175 \| Y \| Y \| 0.99 \| UA, FKH \| \| 13 \| 263 \| ARS1309 \| -4911 \| Y \| Y \| 0.22 \| UA, CEN \| \| 13 \| 287 \| ARS1310 \| 18835 \| Y \| Y \| 0.80 \| UA, CEN \| \| 13 \| 536 \| ARS1320 \| 267629 \| Y \| Y \| 0.88 \| DROMEDARY, FKH \| \| 13 \| 611 \| ARS1323 \| 343291 \| Y \| Y \| 1.00 \| DROMEDARY \| \| 13 \| 635 \| ARS1324 \| 366507 \| Y \| N \|  \| UA \| \| 13 \| 649 \| ARS1325 \| 381339 \| Y \| Y \| 0.59 \| DROMEDARY \| \| 13 \| 815 \| ARS1330 \| 547364 \| Y \| Y \| 0.90 \| CAMEL \| \| 13 \| 837 \| XIII-837 \| 569405 \| Y \| Y \| 1.69 \| UA \| \| 13 \| 898 \| ARS1332 \| 629832 \| Y \| Y \| 0.94 \| UA \| \| 14 \| 90 \| ARS1407 \| -539153 \| Y \| Y \| 1.24 \| DROMEDARY \| \| 14 \| 322 \| ARS1415 \| -306755 \| Y \| Y \| 0.76 \| UA, FKH \| \| 14 \| 546 \| ARS1421 \| -82735 \| Y \| Y \| 1.05 \| UA \| \| 14 \| 561 \| ARS1422 \| -67573 \| Y \| Y \| 0.97 \| CAMEL, FKH \| \| 14 \| 568 \| XIV-568 \| -60451 \| Y \| Y \| 1.04 \| UA \| \| 14 \| 577 \| XIV-577 \| -51818 \| Y \| Y \| 1.20 \| UA \| \| 14 \| 610 \| ARS1424 \| -19236 \| Y \| Y \| 0.29 \| UA, CEN \| \| 14 \| 636 \| ARS1426 \| 6963 \| Y \| N \|  \| UA, CEN \| \| 15 \| 36 \| ARS1506.5 \| -290859 \| Y \| Y \| 0.66 \| DROMEDARY \| \| 15 \| 85 \| ARS1508 \| -241325 \| Y \| Y \| 1.78 \| DROMEDARY \| \| 15 \| 114 \| ARS1509 \| -212681 \| Y \| Y \| 0.93 \| UA, FKH \| \| 15 \| 155 \| ARS1509.5 \| -171427 \| Y \| Y \| 0.98 \| UA \| \| 15 \| 167 \| ARS1510 \| -159547 \| Y \| Y \| 0.87 \| UA, FKH \| \| 15 \| 228 \| ARS1510.5 \| -98845 \| Y \| Y \| 0.66 \| UA, FKH \| \| 15 \| 278 \| ARS1511 \| -48991 \| Y \| Y \| 0.86 \| CAMEL, FKH \| \| 15 \| 309 \| ARS1512 \| -17429 \| Y \| Y \| 0.66 \| CEN  DROMEDARY \| \| 15 \| 337 \| ARS1513 \| 10760 \| Y \| Y \| 0.27 \| CEN  DROMEDARY \| \| 15 \| 348 \| XV-348 \| 21604 \| Y \| Y \| 0.38 \| UA \| \| 15 \| 354 \| XV-354 \| 27671 \| Y \| N \|  \| UA \| \| 15 \| 437 \| ARS1513.5 \| 110205 \| Y \| Y \| 1.10 \| UA \| \| 15 \| 464 \| XV-464 \| 137644 \| Y \| Y \| 0.80 \| DROMEDARY \| \| 15 \| 490 \| ARS1514 \| 163243 \| Y \| Y \| 1.38 \| DROMEDARY, FKH \| \| 15 \| 874 \| ARS1526 \| 547668 \| Y \| Y \| 1.87 \| DROMEDARY \| \| 15 \| 908 \| ARS1528 \| 581769 \| Y \| Y \| 0.52 \| UA \| \| 15 \| 1054 \| ARS1529.5 \| 727052 \| Y \| Y \| 0.80 \| CAMEL, FKH \| \| 16 \| 73 \| ARS1605 \| -482852 \| Y \| Y \| 0.89 \| DROMEDARY \| \| 16 \| 290 \| ARS1614 \| -266419 \| Y \| Y \| 0.96 \| DROMEDARY \| \| 16 \| 385 \| ARS1618 \| -171352 \| Y \| Y \| 1.23 \| DROMEDARY, FKH \| \| 16 \| 418 \| ARS1619 \| -137767 \| Y \| Y \| 1.02 \| UA, FKH \| \| 16 \| 512 \| ARS1621 \| -44233 \| Y \| N \|  \| UA \| \| 16 \| 553 \| XVI-553 \| -2667 \| Y \| N \|  \| UA, CEN \| \| 16 \| 560 \| XVI-560 \| 3621 \| Y \| N \|  \| UA, CEN \| \| 16 \| 564 \| ARS1622 \| 7930 \| Y \| N \|  \| UA \| \| 16 \| 634 \| ARS1623 \| 77981 \| Y \| Y \| 0.58 \| DROMEDARY \| \| 16 \| 685 \| ARS1624 \| 128496 \| Y \| Y \| 1.19 \| DROMEDARY \| \| 16 \| 777 \| ARS1626.5 \| 221025 \| Y \| Y \| 0.86 \| CAMEL, FKH \| \| 16 \| 819 \| ARS1627 \| 263261 \| Y \| Y \| 1.00 \| DROMEDARY \| \| 16 \| 843 \| ARS1628 \| 286758 \| Y \| Y \| 0.65 \| UA \| |
| --- | --- | --- | --- | --- | --- | --- | --- | --- | --- | --- | --- | --- | --- | --- | --- | --- | --- | --- | --- | --- | --- | --- | --- | --- | --- | --- | --- | --- | --- | --- | --- | --- | --- | --- | --- | --- | --- | --- | --- | --- | --- | --- | --- | --- | --- | --- | --- | --- | --- | --- | --- | --- | --- | --- | --- | --- | --- | --- | --- | --- | --- | --- | --- | --- | --- | --- | --- | --- | --- | --- | --- | --- | --- | --- | --- | --- | --- | --- | --- | --- | --- | --- | --- | --- | --- | --- | --- | --- | --- | --- | --- | --- | --- | --- | --- | --- | --- | --- | --- | --- | --- | --- | --- | --- | --- | --- | --- | --- | --- | --- | --- | --- | --- | --- | --- | --- | --- | --- | --- | --- | --- | --- | --- | --- | --- | --- | --- | --- | --- | --- | --- | --- | --- | --- | --- | --- | --- | --- | --- | --- | --- | --- | --- | --- | --- | --- | --- | --- | --- | --- | --- | --- | --- | --- | --- | --- | --- | --- | --- | --- | --- | --- | --- | --- | --- | --- | --- | --- | --- | --- | --- | --- | --- | --- | --- | --- | --- | --- | --- | --- | --- | --- | --- | --- | --- | --- | --- | --- | --- | --- | --- | --- | --- | --- | --- | --- | --- | --- | --- | --- | --- | --- | --- | --- | --- | --- | --- | --- | --- | --- | --- | --- | --- | --- | --- | --- | --- | --- | --- | --- | --- | --- | --- | --- | --- | --- | --- | --- | --- | --- | --- | --- | --- | --- | --- | --- | --- | --- | --- | --- | --- | --- | --- | --- | --- | --- | --- | --- | --- | --- | --- | --- | --- | --- | --- | --- | --- | --- | --- | --- | --- | --- | --- | --- | --- | --- | --- | --- | --- | --- | --- | --- | --- | --- | --- | --- | --- | --- | --- | --- | --- | --- | --- | --- | --- | --- | --- | --- | --- | --- | --- | --- | --- | --- | --- | --- | --- | --- | --- | --- | --- | --- | --- | --- | --- | --- | --- | --- | --- | --- | --- | --- | --- | --- | --- | --- | --- | --- | --- | --- | --- | --- | --- | --- | --- | --- | --- | --- | --- | --- | --- | --- | --- | --- | --- | --- | --- | --- | --- | --- | --- | --- | --- | --- | --- | --- | --- | --- | --- | --- | --- | --- | --- | --- | --- | --- | --- | --- | --- | --- | --- | --- | --- | --- | --- | --- | --- | --- | --- | --- | --- | --- | --- | --- | --- | --- | --- | --- | --- | --- | --- | --- | --- | --- | --- | --- | --- | --- | --- | --- | --- | --- | --- | --- | --- | --- | --- | --- | --- | --- | --- | --- | --- | --- | --- | --- | --- | --- | --- | --- | --- | --- | --- | --- | --- | --- | --- | --- | --- | --- | --- | --- | --- | --- | --- | --- | --- | --- | --- | --- | --- | --- | --- | --- | --- | --- | --- | --- | --- | --- | --- | --- | --- | --- | --- | --- | --- | --- | --- | --- | --- | --- | --- | --- | --- | --- | --- | --- | --- | --- | --- | --- | --- | --- | --- | --- | --- | --- | --- | --- | --- | --- | --- | --- | --- | --- | --- | --- | --- | --- | --- | --- | --- | --- | --- | --- | --- | --- | --- | --- | --- | --- | --- | --- | --- | --- | --- | --- | --- | --- | --- | --- | --- | --- | --- | --- | --- | --- | --- | --- | --- | --- | --- | --- | --- | --- | --- | --- | --- | --- | --- | --- | --- | --- | --- | --- | --- | --- | --- | --- | --- | --- | --- | --- | --- | --- | --- | --- | --- | --- | --- | --- | --- | --- | --- | --- | --- | --- | --- | --- | --- | --- | --- | --- | --- | --- | --- | --- | --- | --- | --- | --- | --- | --- | --- | --- | --- | --- | --- | --- | --- | --- | --- | --- | --- | --- | --- | --- | --- | --- | --- | --- | --- | --- | --- | --- | --- | --- | --- | --- | --- | --- | --- | --- | --- | --- | --- | --- | --- | --- | --- | --- | --- | --- | --- | --- | --- | --- | --- | --- | --- | --- | --- | --- | --- | --- | --- | --- | --- | --- | --- | --- | --- | --- | --- | --- | --- | --- | --- | --- | --- | --- | --- | --- | --- | --- | --- | --- | --- | --- | --- | --- | --- | --- | --- | --- | --- | --- | --- | --- | --- | --- | --- | --- | --- | --- | --- | --- | --- | --- | --- | --- | --- | --- | --- | --- | --- | --- | --- | --- | --- | --- | --- | --- | --- | --- | --- | --- | --- | --- | --- | --- | --- | --- | --- | --- | --- | --- | --- | --- | --- | --- | --- | --- | --- | --- | --- | --- | --- | --- | --- | --- | --- | --- | --- | --- | --- | --- | --- | --- | --- | --- | --- | --- | --- | --- | --- | --- | --- | --- | --- | --- | --- | --- | --- | --- | --- | --- | --- | --- | --- | --- | --- | --- | --- | --- | --- | --- | --- | --- | --- | --- | --- | --- | --- | --- | --- | --- | --- | --- | --- | --- | --- | --- | --- | --- | --- | --- | --- | --- | --- | --- | --- | --- | --- | --- | --- | --- | --- | --- | --- | --- | --- | --- | --- | --- | --- | --- | --- | --- | --- | --- | --- | --- | --- | --- | --- | --- | --- | --- | --- | --- | --- | --- | --- | --- | --- | --- | --- | --- | --- | --- | --- | --- | --- | --- | --- | --- | --- | --- | --- | --- | --- | --- | --- | --- | --- | --- | --- | --- | --- | --- | --- | --- | --- | --- | --- | --- | --- | --- | --- | --- | --- | --- | --- | --- | --- | --- | --- | --- | --- | --- | --- | --- | --- | --- | --- | --- | --- | --- | --- | --- | --- | --- | --- | --- | --- | --- | --- | --- | --- | --- | --- | --- | --- | --- | --- | --- | --- | --- | --- | --- | --- | --- | --- | --- | --- | --- | --- | --- | --- | --- | --- | --- | --- | --- | --- | --- | --- | --- | --- | --- | --- | --- | --- | --- | --- | --- | --- | --- | --- | --- | --- | --- | --- | --- | --- | --- | --- | --- | --- | --- | --- | --- | --- | --- | --- | --- | --- | --- | --- | --- | --- | --- | --- | --- | --- | --- | --- | --- | --- | --- | --- | --- | --- | --- | --- | --- | --- | --- | --- | --- | --- | --- | --- | --- | --- | --- | --- | --- | --- | --- | --- | --- | --- | --- | --- | --- | --- | --- | --- | --- | --- | --- | --- | --- | --- | --- | --- | --- | --- | --- | --- | --- | --- | --- | --- | --- | --- | --- | --- | --- | --- | --- | --- | --- | --- | --- | --- | --- | --- | --- | --- | --- | --- | --- | --- | --- | --- | --- | --- | --- | --- | --- | --- | --- | --- | --- | --- | --- | --- | --- | --- | --- | --- | --- | --- | --- | --- | --- | --- | --- | --- | --- | --- | --- | --- | --- | --- | --- | --- | --- | --- | --- | --- | --- | --- | --- | --- | --- | --- | --- | --- | --- | --- | --- | --- | --- | --- | --- | --- | --- | --- | --- | --- | --- | --- | --- | --- | --- | --- | --- | --- | --- | --- | --- | --- | --- | --- | --- | --- | --- | --- | --- | --- | --- | --- | --- | --- | --- | --- | --- | --- | --- | --- | --- | --- | --- | --- | --- | --- | --- | --- | --- | --- | --- | --- | --- | --- | --- | --- | --- | --- | --- | --- | --- | --- | --- | --- | --- | --- | --- | --- | --- | --- | --- | --- | --- | --- | --- | --- | --- | --- | --- | --- | --- | --- | --- | --- | --- | --- | --- | --- | --- | --- | --- | --- | --- | --- | --- | --- | --- | --- | --- | --- | --- | --- | --- | --- | --- | --- | --- | --- | --- | --- | --- | --- | --- | --- | --- | --- | --- | --- | --- | --- | --- | --- | --- | --- | --- | --- | --- | --- | --- | --- | --- | --- | --- | --- | --- | --- | --- | --- | --- | --- | --- | --- | --- | --- | --- | --- | --- | --- | --- | --- | --- | --- | --- | --- | --- | --- | --- | --- | --- | --- | --- | --- | --- | --- | --- | --- | --- | --- | --- | --- | --- | --- | --- | --- | --- | --- | --- | --- | --- | --- | --- | --- | --- | --- | --- | --- | --- | --- | --- | --- | --- | --- | --- | --- | --- | --- | --- | --- | --- | --- | --- | --- | --- | --- | --- | --- | --- | --- | --- | --- | --- | --- | --- | --- | --- | --- | --- | --- | --- | --- | --- | --- | --- | --- | --- | --- | --- | --- | --- | --- | --- | --- | --- | --- | --- | --- | --- | --- | --- | --- | --- | --- | --- | --- | --- | --- | --- | --- | --- | --- | --- | --- | --- | --- | --- | --- | --- | --- | --- | --- | --- | --- | --- | --- | --- | --- | --- | --- | --- | --- | --- | --- | --- | --- | --- | --- | --- | --- | --- | --- | --- | --- | --- | --- | --- | --- | --- | --- | --- | --- | --- |

1. Chromosomal position (kbp).

2. Distance from *ORI* to *CEN* midpoints (bp). Negative distances indicate *ORI*s to left of *CEN*.

3,4. Y indicates *ORI*s that satisfied firing criteria. 169 unchecked *ORI*s fired in WT, 149 fired in *dbf4-zn* and 146 fired in both.

5. For *ORI*s that fired in both WT and *dbf4-zn*, AUC ratios were determined by dividing the *dbf4-zn* ssDNA AUC by the WT ssDNA AUC.

6. From among the 146 unchecked *ORI*s that fire in both WT and *dbf4-zn*, the *dbf4-zn* dataset was assessed for *ORIs* that could readily be assigned to either camel (29) or dromedary (49) profiles. This assignment was made based on whether they demonstrated a split ssDNA peak (camel) or single ssDNA peak (dromedary). *ORI*s that could not be readily placed in either category have been marked as unassigned (UA), with unassigned strictly meaning unassigned as either a camel or dromedary *ORI*. We also examined whether camel or dromedary assignments corresponded with two other previously defined *ORI* attributes, namely whether *ORI*s utilize the *CEN*-dependent (CEN) or Fkh1/2-dependent (FKH) pathways for DDK recruitment. 35 *ORI*s in the table residing +/- 20 kbp from a *CEN* are assigned as *CEN* *ORI*s. FKH ORIs are assigned based on the analysis in Figure 4C.

**Supplementary Table 2:** AUC and ssDNA/kbp values for all meta-profile composites.

|  |  |  | **MIN-MIN** | **INF-INF** |
| --- | --- | --- | --- | --- |
| **COMPOSITE^1^** | ***ORI* GROUP^2^** | **AUC^3^** | **ssDNA/kbp^4^** | **ssDNA/kbp^5^** |
| WT^6^ | UNCH | 299623 | 0.028 | 0.029 |
| *dbf4-zn* | UNCH | 223521 | 0.025 | 0.078 |
| *rad53* 1 | UNCH | 410997 | 0.071 | 0.087 |
| *rad53* 2 | UNCH | 486623 | 0.093 | 0.119 |
| *rad53 dbf4-zn* 1 | UNCH | 54152 | 0.005 | 0.009 |
| *rad53 dbf4-zn* 2 | UNCH | 38493 | 0.005 | 0.009 |
| *rad53 dbf4-D3* | UNCH | 283021 | 0.043 | 0.063 |
| *PRI1* | UNCH | 33355 | 0.037 | 0.038 |
| *pri1-M4* | UNCH | 327721 | 0.074 | 0.139 |
| *rad53* 1 | CH | 182317 | 0.092 | 0.093 |
| *rad53* 2 | CH | 197535 | 0.114 | 0.118 |
| *rad53 dbf4-zn* 1 | CH | 53616 | 0.016 | 0.022 |
| *rad53 dbf4-zn* 2 | CH | 31808 | 0.008 | 0.015 |
| *rad53 dbf4-D3* | CH | 153649 | 0.058 | 0.075 |
| *PRI1* | CH | 11938 | 0.014 | 0.020 |
| *pri1-M4* | CH | 1525 | 0.022 | 0.027 |
| WT | CAMEL | 1031995 | 0.070 | 0.076 |
| *dbf4-zn* | CAMEL | 799548 | 0.052 | 0.054 |
| *rad53* 1 | CAMEL | 1053020 | 0.098 | 0.096 |
| *pri1-M4* | CAMEL | 1087867 | 0.179 | 0.219 |
| WT | DROM | 340427 | 0.027 | 0.030 |
| *dbf4-zn* | DROM | 227556 | 0.040 | 0.101 |
| *rad53* 1 | DROM | 652019 | 0.093 | 0.108 |
| *pri1-M4* | DROM | 246378 | 0.045 | 0.095 |

1. Composite refers to the meta-analysis profiles for different groups of *ORI*s in different mutants.

2. UNCH, early firing unchecked *ORI*s; CH, late firing, Rad53 checked *ORIs*; DROM, dromedary *ORI*s.

3. AUC, area under the curve. Evaluated between MIN left and MIN right values from *ORI* center.

4. Amplitude/base ratios determined using minimum ssDNA values on either side of the *ORI* to determine the base for the profile.

5. Amplitude/base ratios determined using inflection points on either side of the *ORI* to define the base of the profile. This approach deconvolves the profile to identify specific regions of *ORI* ssDNA. See Materials and Methods for details.

6. Similarly shaded rows indicate strains evaluated within the same experiment.

| **Supplemental Table 3.** Number and percentage of Camel (29) and Dromedary (49) ORIs associated with genomic features. | | |  |
| --- | --- | --- | --- |
| **Chr markers (#)** | **#Camel ORIs with marker <20 kb away (%)** | **#Dromedary ORIs with marker <20 kb away (%)** |  |
| Ty&LTRs (940)^1^ | 19 (66%) | 29 (59%) |  |
| tRNAs (298)^1^ | 21 (72%) | 37 (76%) |  |
| DRIP-rnh1rnh2 (778)^2^ | 29 (100%) | 42 (86%) |  |
| Transcription unit head-on with ORI^3^ | 20 (69%) | 34 (69%) |  |

^1^Voytas and Boeke 1993

^2^Costantino and Koshland 2018

^3^Hoffman et al 2015

**Supplemental Figure 1.** Replication bubble structures relevant to the interpretation of the dromedary ssDNA profile. See text for citations. Thick grey lines represent ssDNA on template strands. Green and red lines represent newly synthesized Crick and Watson strands, respectively. Arrows indicate the direction of synthesis. Discontinuous lagging strands are indicated by short arrows. Dashed green and red lines indicate strands degraded by nuclease digestion. Anticipated ssDNA profiles are depicted beneath each structure. Red boxes indicate bubble structures predicted to form a dromedary ssDNA profile and be visualized as hemi-replicated by electron microscopy in both *rad53* + HU and *pri1-M4* mutants.

**(A)** WT in HU. In response to nucleotide depletion, 100 bp regions of ssDNA become exposed on the lagging strand template (dark grey) at forks from early firing, unchecked ORIs, providing the signal to activate the S phase checkpoint.

**(B)** *pri1-M4* mutants accumulate gapped replication bubbles, reflecting reduced initiation of lagging strand synthesis. Leading strand synthesis continues in an uncoupled manner, exposing ssDNA on the lagging strand template. The extent of the gap would be expected to vary, with most gaps expected to still be sufficiently separated to generate a split peak ssDNA profile. In the diagram, ssDNA is depicted as symmetrically arising at both forks, but a mixture of symmetrically and asymmetric gapped bubbles would be expected to occur in different cells.

**(C)** Gapped replication bubbles have been shown to arise in *rad53* mutants treated with HU because of a defect where CMG advances beyond the site of leading strand synthesis. Lagging strand synthesis continues in an uncoupled manner, exposing ssDNA gaps on the leading strand template. As with (B), the extent of these gaps is expected to vary, and both symmetrically and asymmetrically gapped molecules would be predicted to be present in the cell population. Most ssDNA gaps arising from this defect would be expected to produce a split peak ssDNA profile.

**(D)** *pri1-M4* mutants, and possibly also *rad53*+HU cells, have been proposed to generate completely gapped bubbles reflecting a failure to initiate lagging strand synthesis at either or both replication forks. If most firing events in the cell population produced such structures, it would generate a single-peaked ssDNA profile. However, without further nuclease processing, such extensively gapped structures would still not be visualized as the hemi-replicated bubbles that have been shown to be present in both *pri1-M4* and *rad53* + HU cells.

**(E)** Exonuclease resection of nascent strands (dashed red and green lines) from the lagging strand gaps shown in B. The extent of resection could be variable; complete resection is depicted to illustrate how hemi-replicated bubbles could arise due to Exo1 digestion, assuming RNA primers were removed and lagging strand synthesis tracks ligated together. Leading strands gaps in (D) could be resected similarly. Fork reversals would be anticipated to facilitate Exo1 access by exposing nascent 5’ ends. Dashed grey lines indicate complementary ssDNA regions of template strands that would re-anneal and not contribute to the ssDNA profile. Thus, complete resection of both nascent strands on a bubble would not contribute to the ssDNA profile. However, a mixture of resected nascent Watson or Crick strands would produce a single-peaked ssDNA profile and would be revealed as hemi-replicated bubbles by EM.

**(F)** Unidirectional forks. A more severe form of the initiation defect shown in (D) is that only one fork emanates from an *ORI*, accompanied by a failure to initiate lagging strand synthesis. Such unidirectional fork defects have been proposed to occur in both *pri1-M4* and *rad53* + HU mutants. If such a defect equally affected left or right forks, it would directly produce both a dromedary ssDNA profile and hemi-replicated bubbles without Exo1 processing.

**Supplemental Figure 2.** **Camel *ORI*s**. Individual ssDNA replication profiles were generated from the WT+HU and *dbf4-zn*+HU datasets for all 29 camel *ORIs* identified in this study. Camel composite profiles were generated by averaging this set of profiles.

**Supplemental Figure 3.** **Dromedary *ORI*s**. Individual ssDNA replication profiles were generated from the WT+HU and *dbf4-zn*+HU datasets for all 49 dromedary *ORIs* identified in this study. Dromedary composite profiles were generated by averaging the individual profiles.
