# Supplementary figures and images for: The yeast Dbf4 Zn2+ finger domain suppresses single-stranded DNA at replication forks initiated from a subset of origins
Source: Curr Genet. 2022 Feb 11;68(2):253–65. doi: 10.1007/s00294-022-01230-6 (PMC8976809; doi:10.1007/s00294-022-01230-6)

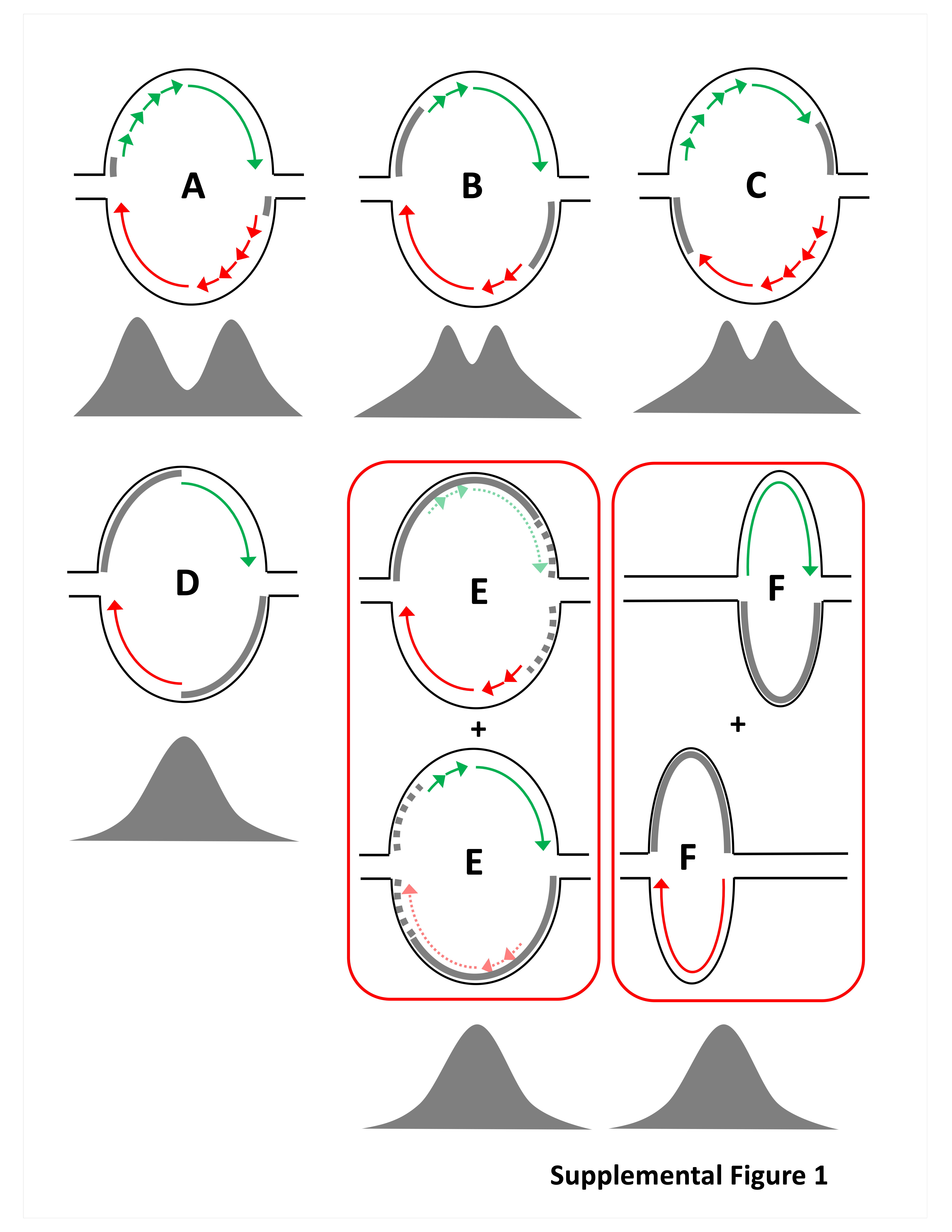

Supplement: Supplementary file 2 — Supplementary file2 (TIFF 2024 KB) [file 294_2022_1230_MOESM2_ESM.tiff]

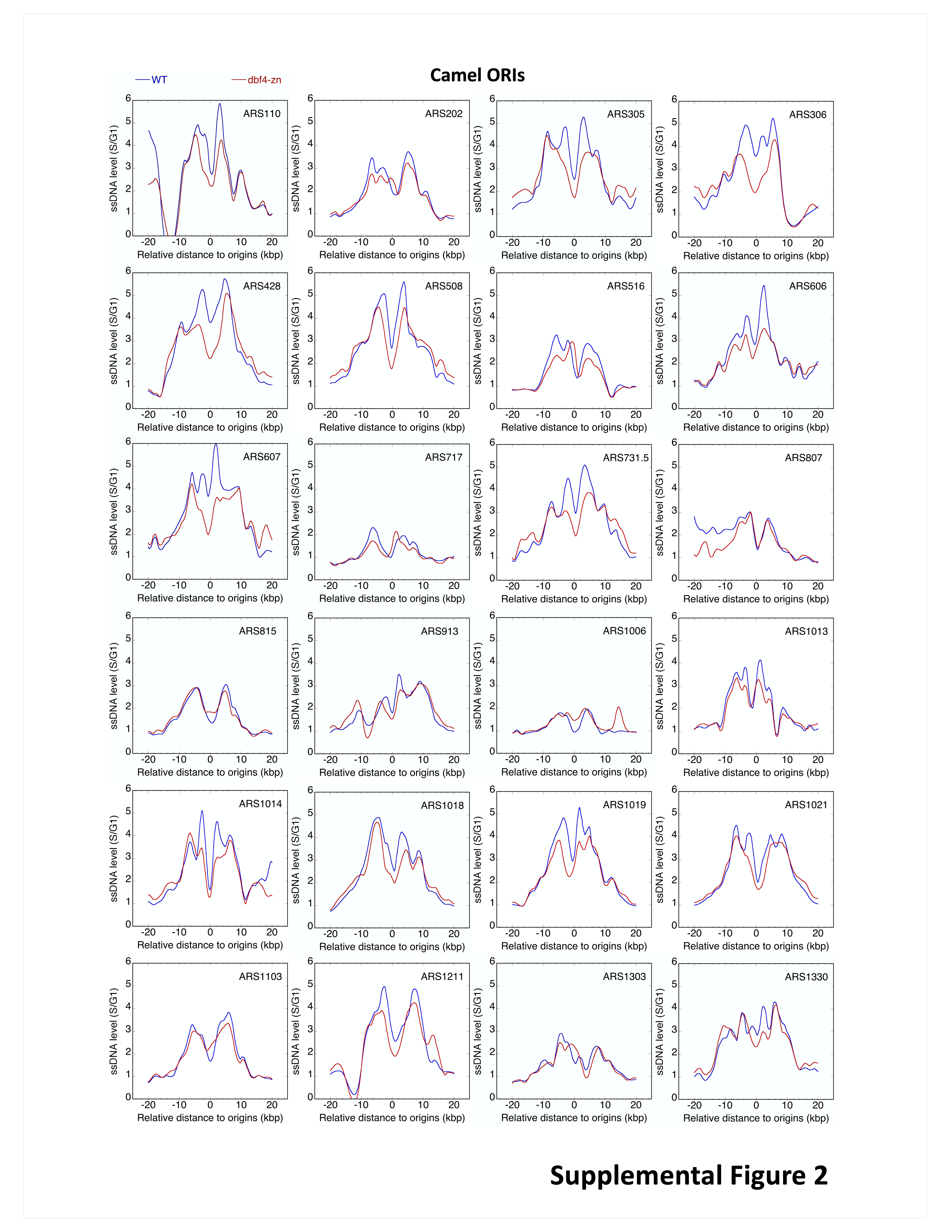

Supplement: Supplementary file 3 — Supplementary file3 (TIFF 9831 KB) [file 294_2022_1230_MOESM3_ESM.tiff]

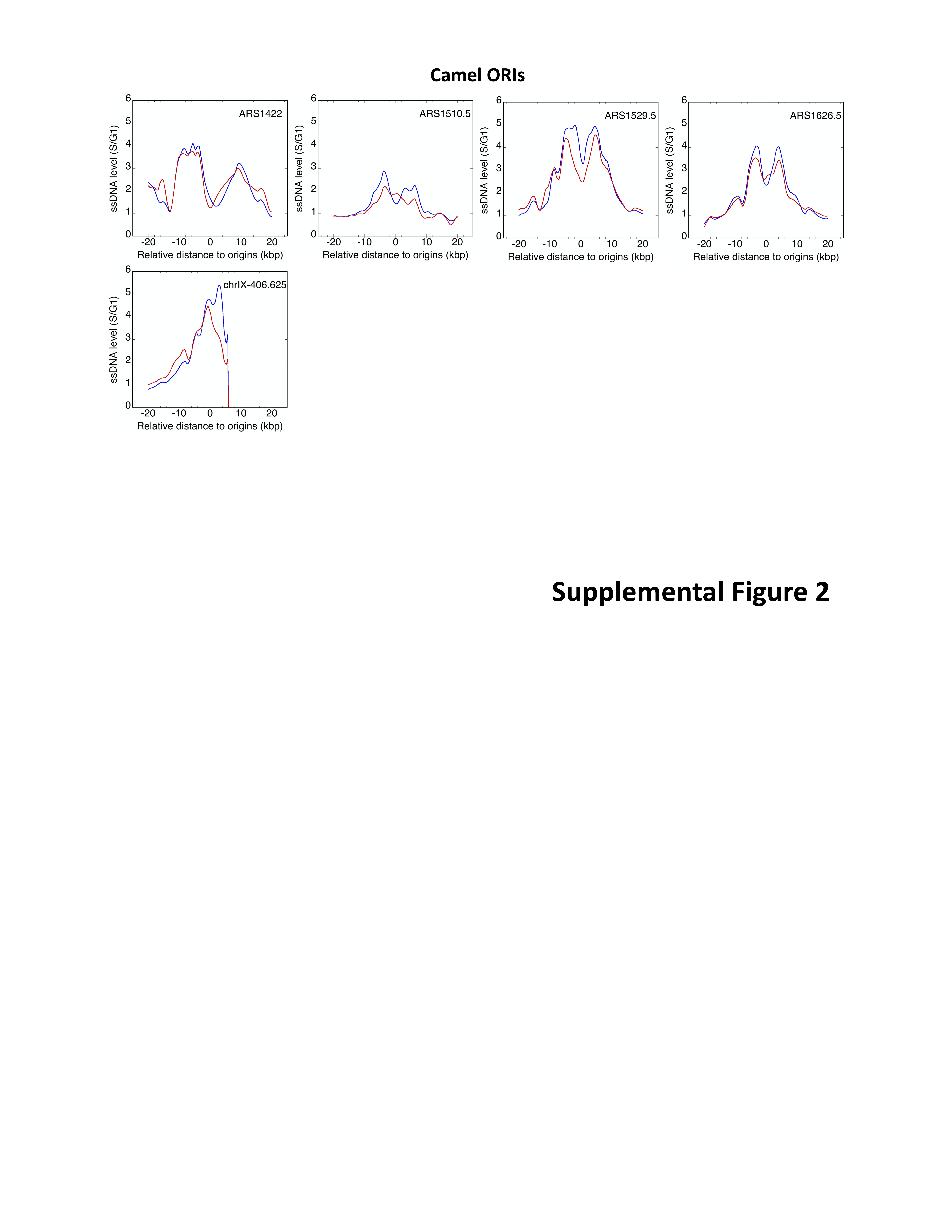

Supplement: Supplementary file 4 — Supplementary file4 (TIFF 2786 KB) [file 294_2022_1230_MOESM4_ESM.tiff]

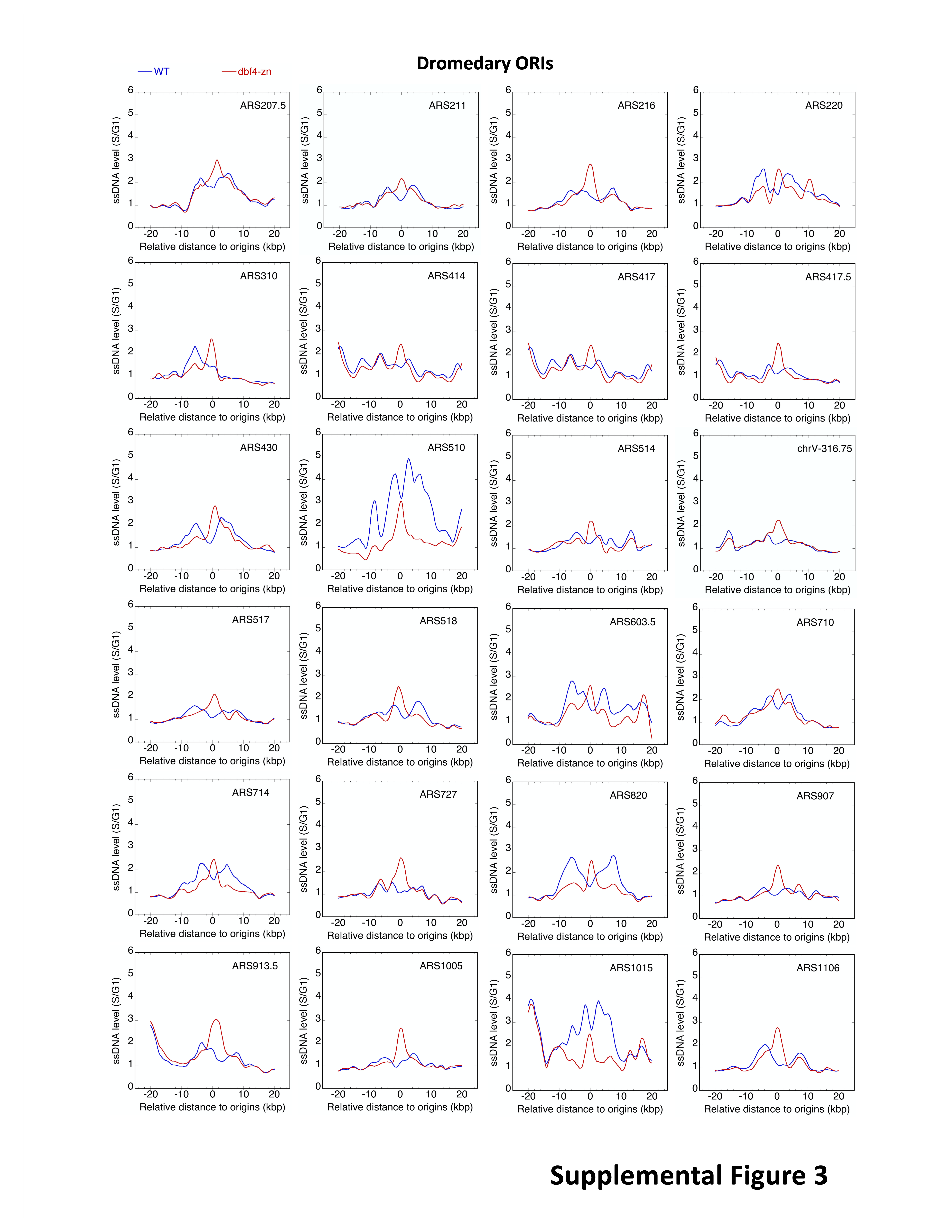

Supplement: Supplementary file 5 — Supplementary file5 (TIFF 8522 KB) [file 294_2022_1230_MOESM5_ESM.tiff]

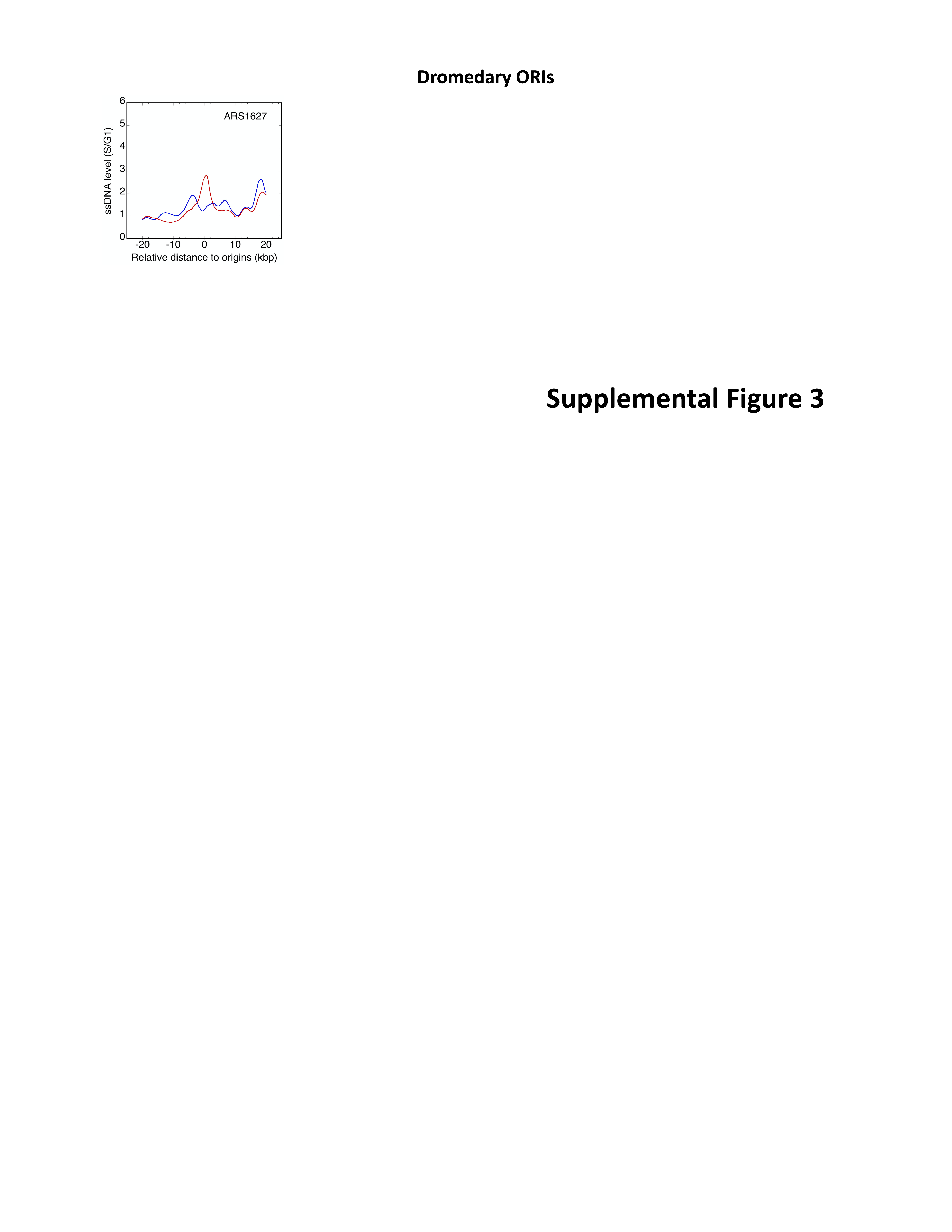

Supplement: Supplementary file 6 — Supplementary file6 (TIFF 1336 KB) [file 294_2022_1230_MOESM6_ESM.tiff]
